# Spatial and Temporal Patterns of Typhoid and Paratyphoid Fever Outbreaks: A Worldwide Review, 1990–2018

**DOI:** 10.1093/cid/ciz705

**Published:** 2019-10-30

**Authors:** Samuel Kim, Kang Sung Lee, Gi Deok Pak, Jean-Louis Excler, Sushant Sahastrabuddhe, Florian Marks, Jerome H Kim, Vittal Mogasale

**Affiliations:** 1 International Vaccine Institute, Seoul, Republic of Korea; 2 Imperial College London, United Kingdom; 3 Department of Medicine, University of Cambridge, Cambridge, United Kingdom

**Keywords:** typhoid, paratyphoid, outbreaks, spatial patterns, review

## Abstract

**Background:**

Analyses of the global spatial and temporal distribution of enteric fever outbreaks worldwide are important factors to consider in estimating the disease burden of enteric fever disease burden.

**Methods:**

We conducted a global literature review of enteric fever outbreak data by systematically using multiple databases from 1 January 1990 to 31 December 2018 and classified them by time, place, diagnostic methods, and drug susceptibility, to illustrate outbreak characteristics including spatial and temporal patterns.

**Results:**

There were 180 940 cases in 303 identified outbreaks caused by infection with *Salmonella enterica* serovar Typhi (*S*. Typhi) and *Salmonella enterica* serovar Paratyphi A or B (*S*. Paratyphi). The size of outbreak ranged from 1 to 42 564. Fifty-one percent of outbreaks occurred in Asia, 15% in Africa, 14% in Oceania, and the rest in other regions. Forty-six percent of outbreaks specified confirmation by blood culture, and 82 outbreaks reported drug susceptibility, of which 54% had multidrug-resistant pathogens. Paratyphoid outbreaks were less common compared to typhoid (22 vs 281) and more prevalent in Asia than Africa. Risk factors were multifactorial, with contaminated water being the main factor.

**Conclusions:**

Enteric fever outbreak burden remains high in endemic low- and middle-income countries and, despite its limitations, outbreak data provide valuable contemporary evidence in prioritizing resources, public health policies, and actions. This review highlights geographical locations where urgent attention is needed for enteric fever control and calls for global action to prevent and contain outbreaks.

Typhoid and paratyphoid fever are potentially severe and life-threatening febrile illnesses caused by *Salmonella enterica* serovar Typhi and *Salmonella enterica* serovar Paratyphi A and B, respectively, collectively known as enteric fever. Despite a dramatic decline in incidence in the early 20th century due to improved sanitation and hygiene practices, enteric fever remains a pressing burden for low- and middle-income countries (LMICs) [1].

Accurate estimation of enteric fever incidence is an epidemiological challenge [2]. Most health facility–based studies underestimate the true incidence, especially in countries where fewer people have access to healthcare services or in which many people seek care outside of the public health system. Lack of rapid and reliable diagnostic methods adds to the difficulty, as blood culture is time- and resource-intensive, misses at least 39% of cases, and is often not used in enteric fever confirmation in developing countries [3]. This problem is exacerbated during outbreaks where the demand on health services may often outstrip the available capacity for culture confirmation [4]. Moreover, reporting systems in these settings often do not capture enteric fever cases rigorously—or in many cases not at all—due to diagnostic or systemic limitations, resulting in further underestimation in country reports [2, 5–8].

Global disease burden estimates depend on extrapolation of available incidence data from published community-based studies that cover well-defined, geographically limited areas [9–12]. These community-based studies are often carried out for a short duration, are resource-intensive, and do not capture concurrent enteric fever outbreaks occurring outside the small study area. Omission of such outbreak data in global disease burden (incidence) estimates results in underestimation of the total burden [11]. The number, size, and location of enteric fever outbreaks worldwide may provide a more comprehensive understanding of the epidemic patterns of disease outbreaks and localize areas with higher typhoid disease burden.

Additionally, large typhoid outbreaks may be important targets for reactive vaccination campaigns. However, to date, there has been limited experience with use of vaccine amid typhoid outbreaks. A better understanding of the size, duration, geographical spread, and age distributions of outbreaks would help inform strategies for vaccine introduction to prevent or contain outbreaks.

Previous reviews on enteric fever outbreaks have focused on local issues and socioeconomic aspects at the national context but not at global levels [13–17]. To address these knowledge gaps, we reviewed outbreaks over the last 27 years to characterize the global spatial and temporal distribution of enteric fever outbreaks and their risk factors.

## METHODS

### Data Sources and Search Strategy

For enteric fever outbreak data, we consulted medical literature databases Medline and Embase as well as the epidemiology-specific databases Global Infectious Disease and Epidemiology Network (GIDEON) and ProMED-mail (the Program for Monitoring Emerging Diseases, an internet-based reporting system on outbreaks of infectious diseases). In the Medline and Embase electronic databases, the following terms were used in the search (“typhoid,” “salmonella,” “enteric fever,” “paratyph*,” “exp Typhoid Fever”) AND (“outbreak*,” “resurgen*,” “re-emergence,” “epidemic*,” “exp Epidemics”). Publications restricted to human studies in the English language from 1 January 1990 to 31 December 2018 were included. Then, outbreaks reported in the GIDEON database (a web-based global infectious diseases database that provides geographical and epidemiological information for infectious disease outbreaks; www.gideononline.com) were reviewed for enteric fever outbreaks in the same time period to identify more articles. We then reviewed ProMED-mail reports from August 1994 to December 2018 using combinations of the key search terms: “typhoid OR *S. typhi* OR salmonella OR salmonellosis OR enteric OR paratyphi OR paratyphoid” (given that all records pertain to outbreaks and in line with the ProMED-mail search guidance) to identify additional reports [18].

Records from the World Health Organization (WHO) Disease Outbreak News reports were cross-referenced for additional outbreaks. Furthermore, the Centers for Disease Control and Prevention’s *Morbidity and Mortality Weekly Report* and the Foodborne Disease Outbreak Surveillance System reports (collects reports of foodborne disease outbreaks from local, state, tribal, and territorial public health agencies) were searched.

Duplicates among the results were removed by identifying unique outbreaks. A standardized approach was used for identifying unique outbreaks in possible duplicate situations (most up-to-date case counts were used). Factors such as proximity of outbreaks in geography, time, and size in the context of the published date of the outbreak and any unique differences (clinical presentation, multidrug resistance, genotype) decided whether a report was a duplicate or unique.

The data on location and GPS (Global Positioning System) of the outbreak, size of outbreak (number affected), case fatality ratio (when available), start and finish dates of the outbreak, diagnostic confirmation of enteric fever, likely cause, limitations, and response to the outbreak were extracted.

For the purpose of this review, we tried to compare enteric fever outbreaks reported by authors to the standard WHO definition of outbreaks as “the occurrence of cases of disease in excess of what would normally be expected in a defined community, geographical area or season. An outbreak may occur in a restricted geographical area, or may extend over several countries. It may last for a few days or weeks, or for several years” [19]. As this comparison was impossible in many study settings, we had to take authors’ reports of outbreak at face value and we defined enteric fever outbreaks as “reported by authors.” Multidrug resistance was defined as an “acquired nonsusceptibility to at least one agent in three or more antimicrobial categories” [20, 21].

Data from outbreak countries were broken down by regions and subregions defined by the United Nations geoscheme and tabulated on a spreadsheet as described in Supplementary Annex 1 and then analyzed using SPSS software (Statistical Package for the Social Sciences) [22]. Methods of the analysis and inclusion criteria were prespecified and presented in Supplementary Annex 1. As a good practice to maintain quality, the literature search and report adhered to the Preferred Reporting Items for Systematic Reviews and Meta-Analyses (PRISMA) statements [23, 24]. The literature review involved 2 reviewers, and the protocol for review is described in Supplementary Annex 1.

## RESULTS

**Figure 1.**
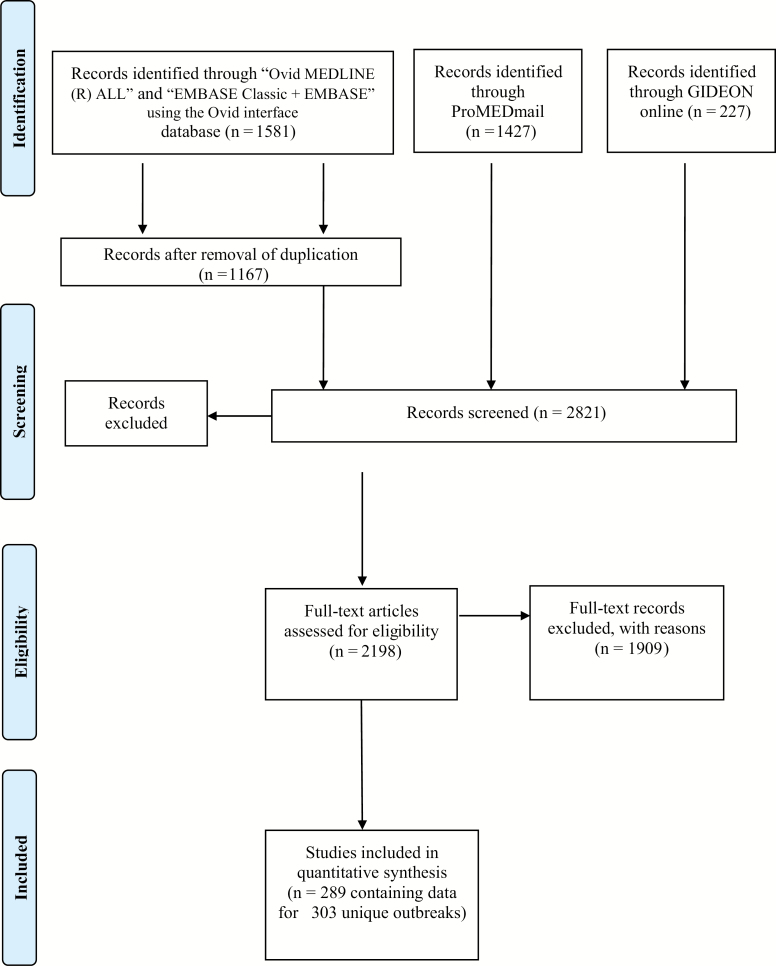
Preferred Reporting Items for Systematic Reviews and Meta-Analyses flowchart for the literature review of enteric fever outbreaks reported from 1 January 1990 to 31 December 2018.

We identified 3235 postings and papers, of which 303 records of unique outbreaks were selected for data extraction (Figure 1). In the process of selection, reports for same outbreaks were merged, duplicates were removed, and abstracts and full texts were screened for inclusion criteria. The main reasons for exclusion were data unavailability on typhoid or paratyphoid fever, not being an outbreak, and not occurring within the period considered. Case fatality ratio and enteric fever complications were not included in the analysis as these data were not readily available for the analysis.

The identified outbreaks varied in size and regions (Supplementary Annex 2) and included 180 940 cases. Of these reported outbreaks, 51% occurred in Asia, followed by Africa (15%) and Oceania (14%) (Table 1). Subregional distribution of outbreaks suggests that South Asia (n = 48) and Southeast Asia (n = 42) share the highest reported burden. Although there were a comparable number of reported outbreaks in Europe (n = 28) and North America (n = 22), the average size of the outbreaks were, however, up to 60 times lower compared to Africa (mean size of 2430 in Africa vs 31 in Europe and 39 North America). India reported the highest number of outbreaks (n = 36), followed by the Philippines (n = 21), Fiji (n = 16), and the United States (n = 16). Six discrete outbreaks were reported in the North Division area of Fiji, a typhoid fever–endemic area. Dushanbe in Tajikistan had 3 discrete outbreaks during the period of 1996–2008 with a size ranging from 100 to 10 677 cases.

**Table 1.** Regional (and Subregional for Asia) Distribution of the Numbers of Outbreaks and Reported Enteric Fever Cases

Regions	No. of Outbreaks	Minimum No. of Reported Cases per Outbreak	Maximum No. of Reported Cases per Outbreak	Sum	Mean	Median	Standard Deviation
Africa	46	3	42 564	111 784	2430	147	7673
Asia	155	1	10 677	62 318	402	79	1348
Central	19	4	10 677	20 478	1078	78	2883
Eastern	22	1	601	2231	106	27	154
Western	20	5	3010	6382	319	50	705
Southern	48	6	5963	22 867	440	101	1373
Southeastern	42	2	3049	10 360	247	77	548
Europe	28	1	277	868	31	15	53
North America	22	1	321	858	39	9	80
South America	7	3	110	159	23	8	39
Central America	4	24	653	857	214	90	295
Oceania	41	2	1200	4096	100	24	215
Total/overall	303	1	42 564	180 940	597	48	3215

**Figure 2.**
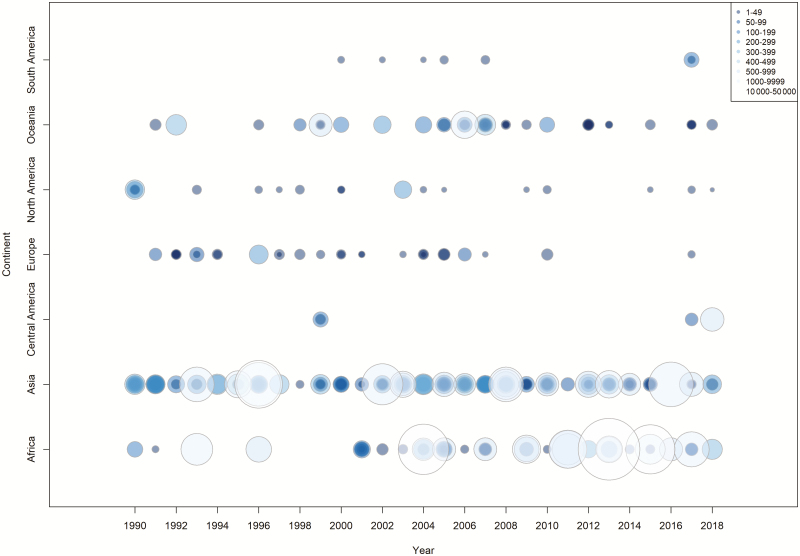
Distribution of enteric fever outbreaks by region and by year for 1990–2018 (each circle represents a discrete outbreak).

**Figure 3.**
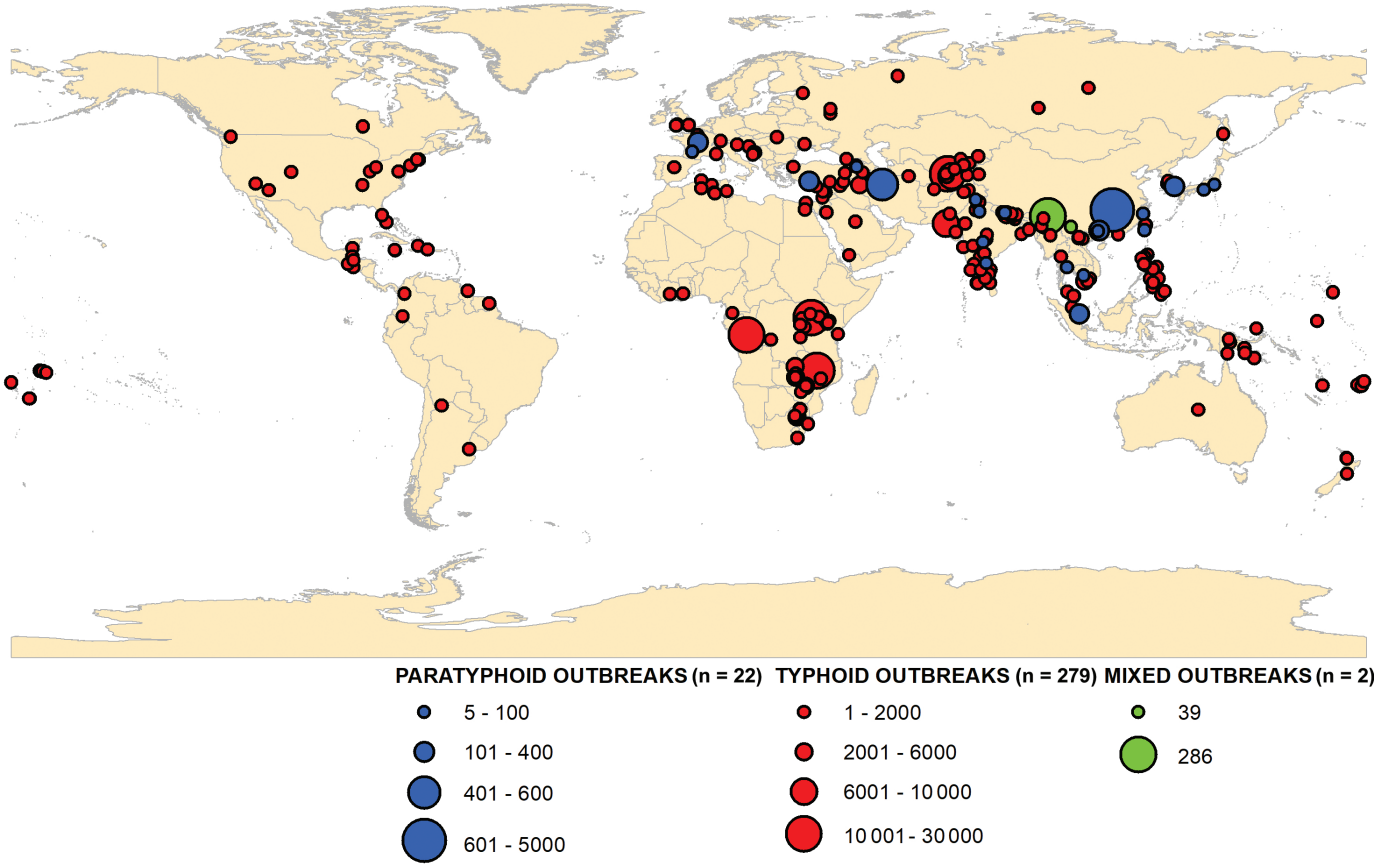
Geographical distribution of typhoid and paratyphoid outbreaks reported from 1 January 1990 to 31 December 2018 (mixed outbreaks: typhoid and paratyphoid).

**Figure 4.**
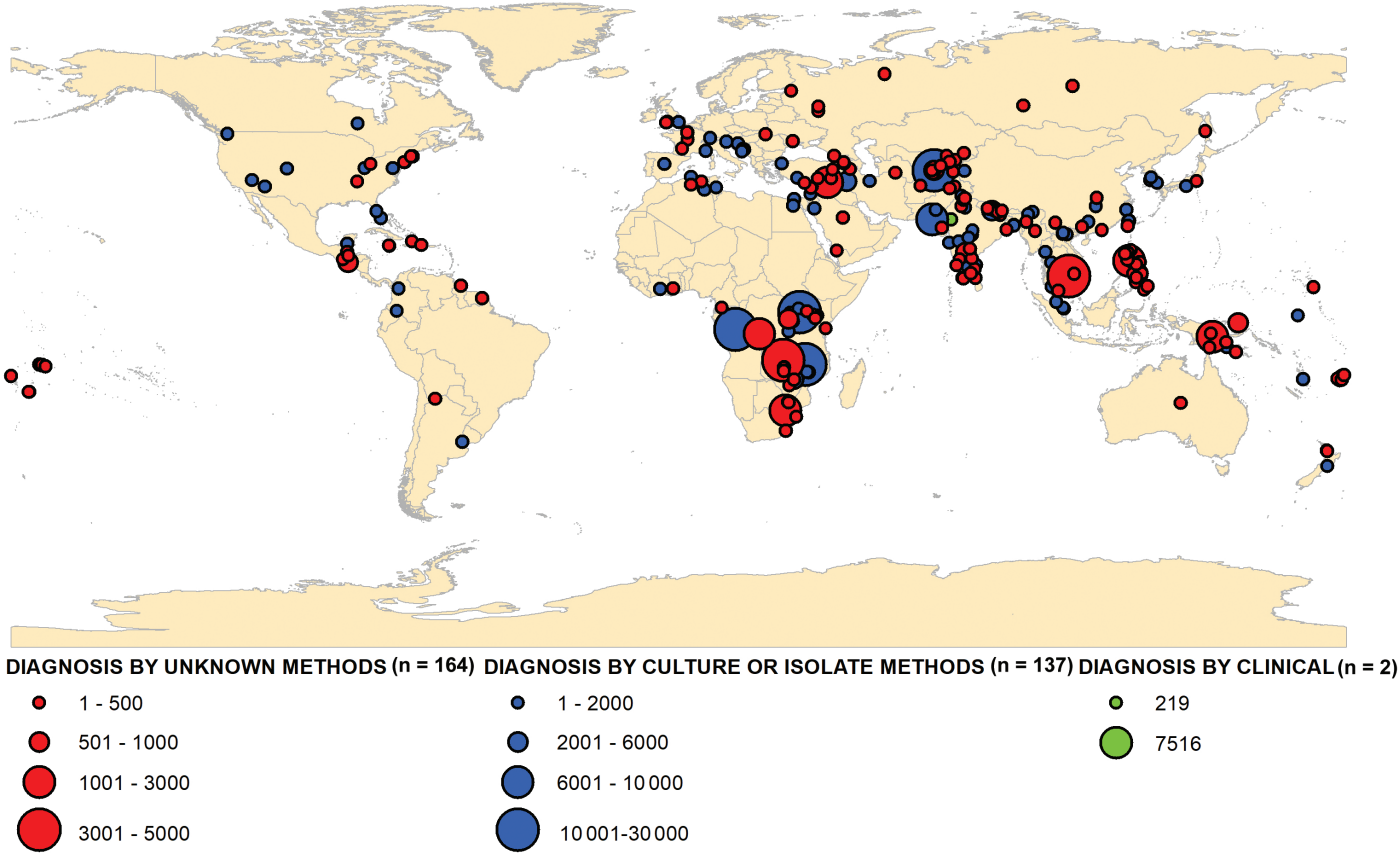
Diagnostic method used for the confirmation of enteric fever outbreaks reported from 1 January 1990 to 31 December 2018.

**Figure 5.**
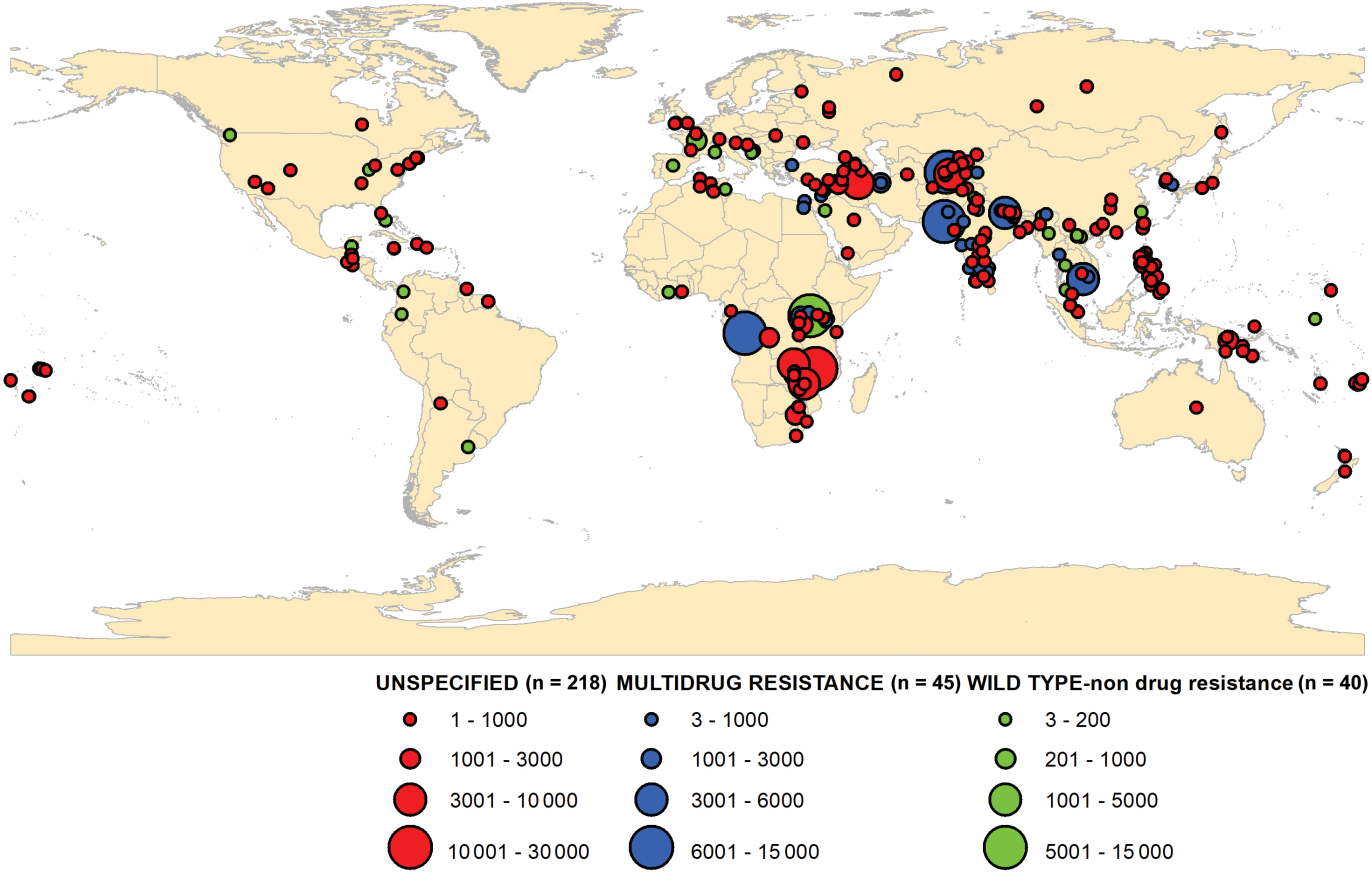
Location of multidrug-resistant strain enteric fever outbreaks reported from 1 January 1990 to 31 December 2018.

There was considerable variability in the number of reported enteric fever outbreaks with a general increasing trend over time with a peak in 2004 (25 cases) and a decreasing trend after 2007 (Figures 2 and 3). There were 281 typhoid fever outbreaks and 22 paratyphoid outbreaks, and the remaining 2 were mixed paratyphoid and typhoid outbreaks. Although typhoid fever outbreaks were equally prevalent between regions, paratyphoid fever outbreaks seemed to occur more frequently in Asia, the Middle East, and Europe (Figure 4). The outbreaks in Africa, the Americas, and Oceania were predominantly typhoid fever. Blood culture was the method of diagnosis reported in 46% of outbreaks included in this study (Figure 5). Of the 303 outbreaks, 137 were confirmed by blood culture and 2 were confirmed by Widal test or clinical diagnosis, but 164 did not report the confirmation method; of those, 85 were “lower-middle income economies” or “low-income economies” (as defined by the World Bank). Forty-five outbreaks involved predominantly multidrug-resistant strains, 40 involved susceptible strains, and 218 reports of outbreaks did not specify the antimicrobial characteristics. Multidrug-resistant strain outbreaks were primarily from Asia, although some occurred in Africa (Figure 5).

### Risk Factors

Of the 303 reported outbreaks, 120 (40%) directly pointed to contaminated water as at least 1 of the associations with the outbreak. Forty-seven outbreaks (16%) were reported to be solely related to foodborne vectors, 9 (3%) were imported from other regions, and 4 were related to person-to-person contact alone. Of the 9 imported outbreaks, the countries of origin included Tajikistan, Nepal, India, and Indonesia. Outbreaks in Europe included 15 (54%) in middle-income (upper-middle-income and lower-income economies) countries such as Russia, Croatia, Ukraine, and Bosnia and Herzegovina, where the outbreaks were attributed to contaminated water associated with war conditions and concurrent breakdown of hygiene and sanitation facilities [25–27]. Other alleged causes included asymptomatic food handlers who were chronic carriers and cases without clear etiology. Five of the 50 European and North American outbreaks were imported [28–31]. A widespread outbreak in the United States during 1998–1999 was linked to the consumption of a tropical fruit prepared in Guatemala and Honduras [32]. Five outbreaks in North America originated in LMICs (Jamaica, Dominican Republic, and Haiti) [33–35]. A single, multistate outbreak associated with sexual transmission was reported in May 2000 in the United States [36].

**Figure 6.**
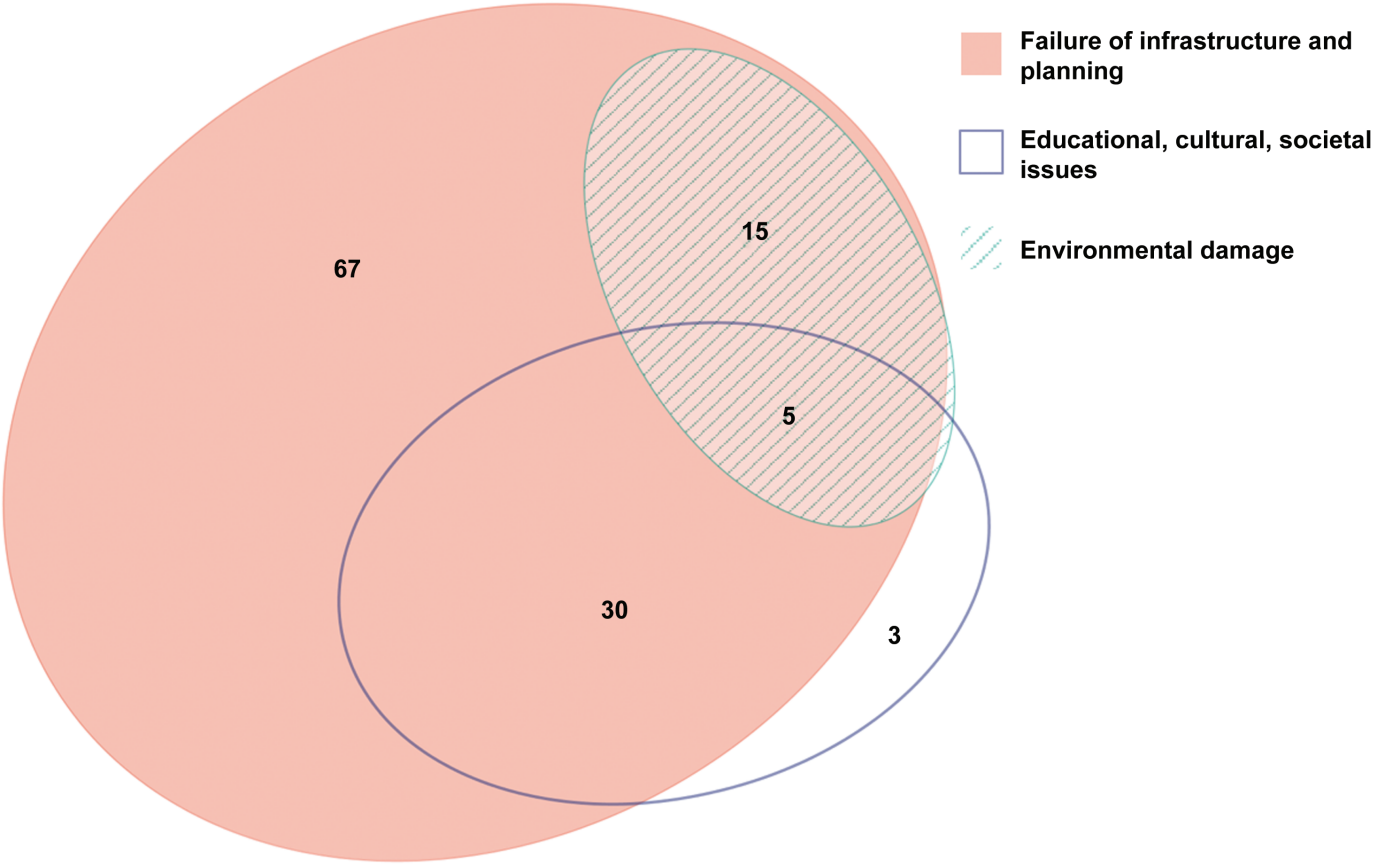
Venn diagram showing risk factors for enteric fever outbreaks reported from 1 January 1990 to 31 December 2018.

Although alleged causes of outbreaks may be multifactorial, risk factors and causes attributed to each reported outbreak linked to contaminated water were classified into 3 broad categories: poor water/sanitation infrastructure and urban planning; environmental damage; and educational, cultural, and societal issues (Table 2; Figure 6). Each outbreak may possibly fall into multiple categories. These broad categories allow the inclusion of the risks factors reported in each outbreak and are consistent with other reviews that examine global outbreaks of infectious diseases [37]. Furthermore, the different categories reflect the varying levels of cost required to ameliorate the risk factors associated with the outbreak. With those caveats, the majority of water-related outbreaks were ascribed to failure of infrastructure or planning (117 outbreaks), whereas 38 outbreaks were attributed to educational, cultural, and societal issues and 20 outbreaks described environmental damage as a contributable risk factor.

**Table 2.** Categorization of Main Risk Factors Associated With Waterborne Enteric Fever Outbreaks Worldwide From 1990 to 2018

Failure in Infrastructure and Planning (n = 117)	Environmental Damage (n = 20)	Educational, Cultural, and Societal Issues (n = 38)
Lack of infrastructure (to provide clean water)	Unseasonal rains, earthquake, flooding	Poor hygiene practices (not washing hands or boiling water)
Proximity of drinking water source to irrigation/sanitation facilities	Civil unrest, war	Garbage dumping
Clean water shortage (due to population growth)	Fall of Soviet period (decrease funds and access to healthcare)	Hiding the problem (to avoid public scare)
Access to healthcare facilities	Antiterrorist operations	Overcrowding, mass gatherings

## DISCUSSION

We identified 303 enteric fever outbreaks worldwide from 1990 to 2018 and showed that the reported number and size of outbreaks are not decreasing but instead growing over time with a burden highest in LMICs where typhoid is endemic. A systematic review and risk-adjusted estimation of burden of typhoid fever using prospective cohort studies in 2014 offers points of comparisons with our global outbreak review [11]. We showed that reported outbreaks overlap the geographical areas that are endemic for typhoid such as Africa and South Asia. Areas with risk factors for outbreaks are also endemic for enteric fever, including the 3 countries with the highest number of enteric fever outbreaks (India, Philippines, and Fiji). Contaminated water was used to adjust risk in global burden estimations and was found to be an important risk factor in this review [11].

Our study also shows that outbreaks do occur in developed countries in North America and Europe, which often have better systems for detection and reporting compared to LMICs. However, the smaller size of the outbreaks suggests that outbreaks may be better controlled and the determinants different in those settings compared to LMICs. As most high-income countries have already achieved good sanitation and improved microbiologic safety of water and food, the implementation of hygiene education and vaccination jointly alongside a thorough investigation to identify causes of the outbreak may minimize the duration and size of outbreaks.

We found that outbreaks often had a multifactorial alleged cause. Failure of infrastructure or planning combined with educational and cultural practices (poor hand-washing practices and using local rivers to wash, clean, and defecate) often amplify the effects of an outbreak. Changes in the local environment such as cyclical seasonal rains and/or ongoing civil unrest can lead to breakdown of public services such as healthcare, clean water supply, and water and sewage systems (Table 2). Griffith et al in their review of global cholera outbreaks report similarities in the variation by subregions and risk factors [37]. The largest identifiable risk factor for cholera outbreaks was contaminated water sources, which was the same for enteric fever outbreaks. Outbreaks of both diseases appear to occur in similar political and sociocultural settings. Most of the risk factors are manageable and avoidable but require substantial human and financial resources to prevent and control outbreaks. Other studies have shown that hygiene programs, access to clean water, and infrastructure can decrease the number of outbreaks and their duration [38–41].

It was reported that globally the incidence of paratyphoid was increasing in many areas [42–44]. Although paratyphoid outbreaks are also increasing, they are still limited in number.

A recent WHO position paper recommends the programmatic use of the new-generation typhoid conjugate vaccine (TCV) and its use for confirmed outbreaks. However, given that TCV does not protect against paratyphoid fever, it raises concerns regarding its ability to control enteric fever in the future and underscores the need for paratyphoid vaccines or bivalent vaccines that cover both diseases [45, 46].

Another concern is that many *S.* Typhi serotypes were found to be multidrug resistant after detailed investigations. Outbreaks linked to multidrug-resistant strains may be better reported and investigated for various reasons, including higher hospitalization and mortality rates [47–49], and this may represent a possible reporting bias. Antimicrobial resistance has been described in endemic populations but more recently also in chronic carriers of typhoid who developed spontaneous drug-resistance mutations in vivo and caused local outbreaks [50–53].

There were some limitations to this work. The WHO definition of an outbreak allows some user discretion and was often used liberally in identified reports. It was difficult to define whether an outbreak in an endemic country was an actual outbreak or whether it was an insignificant variation in an endemic population. One of the key challenges in identifying outbreaks is that most enteric fever–endemic settings lack reliable, precise data on baseline typhoid and paratyphoid incidence. Most enteric fever reporting systems only capture a small fraction of true cases. When increases in observed cases occur, it is difficult to determine whether this is due to an actual increase in typhoid incidence or differences in detection and reporting. This poses a major challenge to identifying outbreaks and underscores the need for improved, sustainable surveillance for enteric fever. The initial high capital cost of improving surveillance capacity may be balanced by the cost savings and health benefits wrought by early detection and accurate outbreak burden identification [54].

A growing and more connected world with increased tourism and travel, further complicated by displacement due to sociopolitical events, can be seen to trigger outbreaks in an endemic area. Genotyping suggests that outbreaks often travel beyond borders and continue as global waves of cases, which is an interconnected outbreak (Table 2) [55, 56]. Added complexity regarding diagnosis and tracking of outbreaks occurs as chromosomal rearrangement can occur within chronic carriers, producing genomic diversity [51, 52].

Several methods have been proposed for public health surveillance that relies on a baseline “normal incidence” within a statistical algorithm. More recently, newer and more sensitive methods have been proposed [32, 57]. The challenge of reliably identifying and tracking outbreaks may be greatly assisted with integration of artificial intelligence to identify statistical irregularity in detected cases especially in endemic areas. As surveillance systems mature globally, the scope to apply these methods can increase, especially as the value of these systems is not mutually exclusive with the value of increasing the surveillance capacity (especially in resource-poor settings). This is further validated by the increasing complexity and capacity of algorithms to incorporate disease trends and behavioral and demographic data [58, 59].

Another limitation is the difference in outbreak detection and confirmation. Outbreak confirmation biological methods varied from Widal test, blood culture, and stool culture to unknown methods; all existing typhoid diagnostics have substantial limitations in accuracy [3]. The Widal test has been shown to be of low specificity for typhoid fever, particularly in endemic settings, and may overestimate the number of cases in typhoid fever outbreaks [60–65]. Typhoid fever is often difficult to confirm by culture outside of the time window (within the first 2 weeks) [61, 66–68] and may result in underestimation. The clinical syndrome of enteric fever is nonspecific and difficult to distinguish from other febrile illnesses, including malaria, viral illnesses, and rickettsia infections; studies have indicated that typhoid is frequently misdiagnosed clinically. An outbreak interpreted as “typhoid outbreak” and reported without laboratory confirmation may or may not be a typhoid outbreak. These challenges highlight the need for standardized reporting of outbreaks to allow consistency of outbreak detection and reporting globally.

Countries with no or poor surveillance systems may have underdetection bias and may have poor sensitivity to detect typhoid outbreaks. By contrast, regions with established methods of surveillance and alert procedures for disease control, which are predominantly high- or upper middle-income countries, may be overrepresented among reported outbreaks. Similarly, outbreaks focusing on multidrug-resistant strains are reported more readily, for example in the United States, which reported 10 outbreaks in the country over the study period, putting it in the top 5 outbreak countries, despite an overall low incidence of waterborne enteric diseases. Since diverse sources and researchers reported the studies, estimating reporting bias over time and space was not possible. Furthermore, only English-language papers were included in this study, which may underestimate outbreaks from non-English-speaking areas, although non-English translated reports were included in the ProMED-mail database.

ProMED-mail provides a real-time, online source of information about outbreaks, but being a passive, non-peer-reviewed monitoring system, it may not be sensitive or specific enough for all enteric fever outbreaks. Furthermore, healthcare access limitations in LMICs may result in underreporting or delayed reporting. Although this review spans from 1990 to 2018, ProMED-mail only became established in 1994, hence the paucity of information prior that period. We found little overlap of outbreaks reported in ProMED-mail with those reported in the scientific literature captured by Medline, suggesting that several outbreaks may not have been reported.

**Figure 7.**
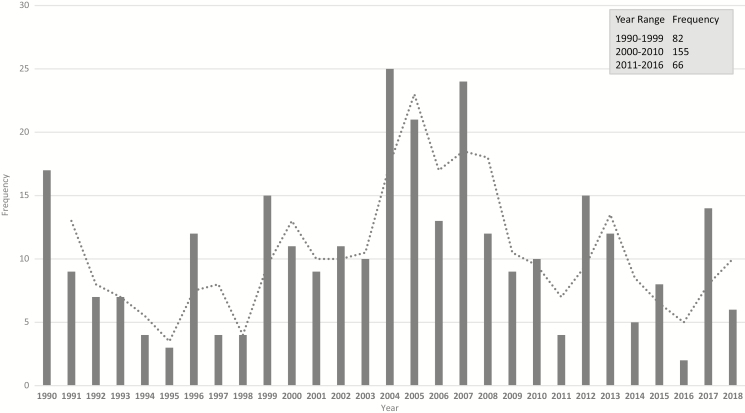
Histogram showing frequency of total enteric fever outbreaks per year and per year range from 1 January 1990 to 31 December 2018 with moving average.

Chan et al quantified global outbreak detection and public reporting (including enteric fever) [69]. They found that the number of total outbreaks and outbreak cases increased dramatically over time from 1980 to 2010 (26 to 106 outbreaks) even when controlling for internet usage (shown to improve detection and reporting). This finding was replicated in this current review, but we also found a trend of decreasing outbreaks after 2007 (Figure 7) [70–72].

## CONCLUSIONS

Enteric fever outbreaks remain common in endemic LMICs and, despite their limitations, outbreak data provide valuable contemporary evidence in prioritizing resources and public health policies and actions. The new-generation TCV is now WHO-prequalified and recommended by WHO for programmatic use both in routine and outbreak settings. Additionally, Gavi, the Vaccine Alliance has approved a funding window to assist countries with investments in TCVs. In this context, enteric fever outbreak mapping provides policy impetus for evidence-based prioritization of TCV introduction. To support such disease control efforts, there is an urgent need to standardize detection, reporting, and monitoring of outbreaks in a consistent manner at the national and international levels.

## Supplementary Data

Supplementary materials are available at *Clinical Infectious Diseases* online. Consisting of data provided by the authors to benefit the reader, the posted materials are not copyedited and are the sole responsibility of the authors, so questions or comments should be addressed to the corresponding author.

ciz705_suppl_Supplementary_Material

## Notes

***Author contributions.*** S. K. conducted the study as primary reviewer, analyzed the data, and wrote the manuscript. V. M. conceptualized and guided the study, served as a second reviewer, and edited and finalized the manuscript. K. S. L. and G. D. P. developed the figures linking to Global Positioning System coordinates. J.-L. E., S. S., F. M., and J. H. K. edited the manuscript and contributed to the analysis. All authors have accepted the final manuscript.

***Financial support.*** This research was funded by the Bill & Melinda Gates Foundation [OPP1127988]. The International Vaccine Institute acknowledges its donors including the Republic of Korea and the Swedish International Development Cooperation Agency. This publication was made possible through a grant from the Bill & Melinda Gates Foundation [OPP1201031].

***Supplement sponsorship.*** This article was published as part of the supplement “Severe Typhoid Fever in Africa (SETA) Program” sponsored by the International Vaccine Institute.

***Potential conflicts of interest.*** J. H. K. has received payments from Takeda as an Scientific Advisory Board consultant. All other authors report no potential conflicts of interest. All authors have submitted the ICMJE Form for Disclosure of Potential Conflicts of Interest. Conflicts that the editors consider relevant to the content of the manuscript have been disclosed.
