# Supplementary material for: Spatial and Temporal Patterns of Typhoid and Paratyphoid Fever Outbreaks: A Worldwide Review, 1990–2018
Source: Clin Infect Dis. 2019 Oct 30;69(Suppl 6):S499–509. doi: 10.1093/cid/ciz705 (PMC6821269; doi:10.1093/cid/ciz705)
Supplement: ciz705_suppl_Supplementary_Material [file ciz705_suppl_supplementary_material.docx]

Supplementary File for Spatial and temporal patterns of typhoid and paratyphoid fever outbreaks: A worldwide systematic review, 1990-2018

**Spatial and temporal patterns of typhoid and paratyphoid fever outbreaks: A worldwide systematic review, 1990-2016**

*Samuel Kim^,1,2^, Vittal Mogasale^1^, Kang Sung Lee^1^, Jean-Louis Excler^1^, Sushant Sahastrabuddhe^1^, Florian Marks^1^, Jerome H. Kim^1^.*

**Affiliations**

^1^International Vaccine Institute, Seoul, South Korea; ^2^Imperial College London, London, United Kingdom

**Corresponding author**

Dr Vittal Mogasale, International Vaccine Institute, Policy and Economic Research Department, SNU Research Park 1 Gwanak-ro, Gwanak-gu, Seoul, Republic of Korea, Tel (82-2) 881 1379, Fax (82-2) 872-2803

Email: [vmogasale@ivi.int](mailto:vmogasale@ivi.int); [vmogasale@gmail.com](mailto:vmogasale@gmail.com)

**Annex 1: Protocol for spatial and temporal patterns of typhoid and paratyphoid fever outbreaks: A worldwide systematic review, 1990- 2016**

**…………………………………………………………………………………………………………**

**Review working group:**

Vittal Mogasale (PER, IVI)

Samuel Kim

**Research question:**

What is the global spatial and temporal distribution of enetric fever outbreaks?

PICOTS: P (Patient): Patient with typhoid

I (Intervention): NA

C (Comparison): NA

O (Outcome): Typhoid fever incidence, case fatality rates and complications

T (Time): 1^st^ Jan 1990 to 31^st^ December 2018

S (Study design): All study types

**Objectives:**

Primary: To map out the distribution and quantify the size of outbreaks of enteric fever

Secondary: To estimate case fatality rates in enteric fever outbreaks.

**Inclusion criteria:**

1. Publications listed from Jan 1, 1990 to 31^st^ December 2018
2. Studies in English language
3. Listed in MEDLINE and/or Embase database and/or ProMED and/or GIDEON database
4. Study designs: All study types included
5. Definition of outbreaks- If an author reported the occurrence of enteric fever as an outbreak in the manuscript, it will be included it in our analysis. We will compare enteric fever outbreak reported by authors to the standard WHO definition of outbreaks as “the occurrence of cases of disease in excess of what would normally be expected in a defined community, geographical area or season”.
6. Diagnostic criteria (confirmed via lab based- blood culture or clinically)

**Exclusion criteria: NA**

**Search Strategy (**see screenshot 1)**:**

- 1. **Typhoid**.mp
  2. **Salmonella typhi**.mp
  3. **Enteric fever**.mp
  4. **Paratyph***.mp
  5. **Outbreak***.mp
  6. **Resurgen***.mp
  7. **Re-emergence**.mp
  8. **Epidemic***.mp
  9. exp **Typhoid Fever**
  10. exp **epidemic**
  11. 1 or 2 or 3 or 4 or 9
  12. 5 or 6 or 7 or 8 or 10
  13. 11 and 12
  14. limit 13 to (Human and English language and Yr="1990 - 2018")

*Search Limits:* Publication between 1^st^ Jan 1990 to 31^st^ December 2018, English language and humans

*Search date:* June 2019

*Review Steps*

Step 1: Type Term 1 to Term 4 in the Ovid^1^ search bar using “Ovid MEDLINE (R) ALL” and “EMBASE Classic + EMBASE” resource. List the total number of papers.

Step 2: Type Term 5 to Term 8 in search bar. List the total number of papers.

Step 3: Using the explode function map out terms “Typhoid fever” and “epidemic”. List the total number of papers.

Step 4: Combine Term 1-4 with exploded term “Typhoid” [Term 11]

Step 5: Combine Term 5-8 with exploded term “Epidemic” [Term 12]

Step 6: Combine Term 11 with Term 12 using “AND” term. List the total number of papers. [Term 13]

Step 7: Apply limits. List the total number of papers. [Term 14]

Step 8: Send to citation manager and import to data management software (End Note)

Step 9: Remove duplicates

Step 10: Additional unique records identified through ProMEDmail^2^ database. Given all records pertain to outbreaks, appropriate search terms were as follows: “typhoid OR s.typhi OR salmonella OR salmonellosis OR enteric OR paratyphi OR paratyphoid” These will be added to the list

Step 11: Additional unique records identified through GIDEON^3^ database (Diseases->Typhoid and enteric fever->Distribution->Outbreaks). These will be added to the list

Step 12: Screen the title and abstract to verify the eligibility based on inclusion and exclusion criteria. List the total number of papers

Step 13: Obtain Full text papers and confirm eligibility. Record reasons for exclusion. List the total number of papers included for qualitative synthesis.

Step 14: List the total number of papers included for quantitative synthesis.

Step 15: Summarize literature review by PRISMA figure below

^1^ <http://ovidsp.tx.ovid.com/>

^2^ http://www.promedmail.org/

^3^ <https://www.gideononline.com/>

A standardized approach was used for identifying unique outbreaks in possible duplicate situations. Factors such as proximity of outbreaks in geography, time and size in the context of the published date of the outbreak and any unique differences (clinical presentation, multi resistance, serovar/ phagetype) decided whether a report was a duplicate or unique.

The search will involve two reviewers. Initial search and data extraction will be done by primary reviewer. Second reviewer will repeat the search independently in medical database and verify search results are correct. Any mismatch between two reviewers will be updated based on consensus agreed between two reviewers. Primary reviewer will independently extract data from 2^nd^ time from final identified papers. Any mismatch between two extractions will be corrected by revising the paper. Second reviewer will verify for data mismatch in 10% of included papers. If mismatch was found in more than 5% of verified papers, duplicate data extraction will be done by second reviewer to compare the data extracted between the reviewers. Any mismatch will be corrected based on consensus agreed between two reviewers

**Screenshot 1**: search strategy using Ovid interface with MEDLINE resource and EMBASE resource.


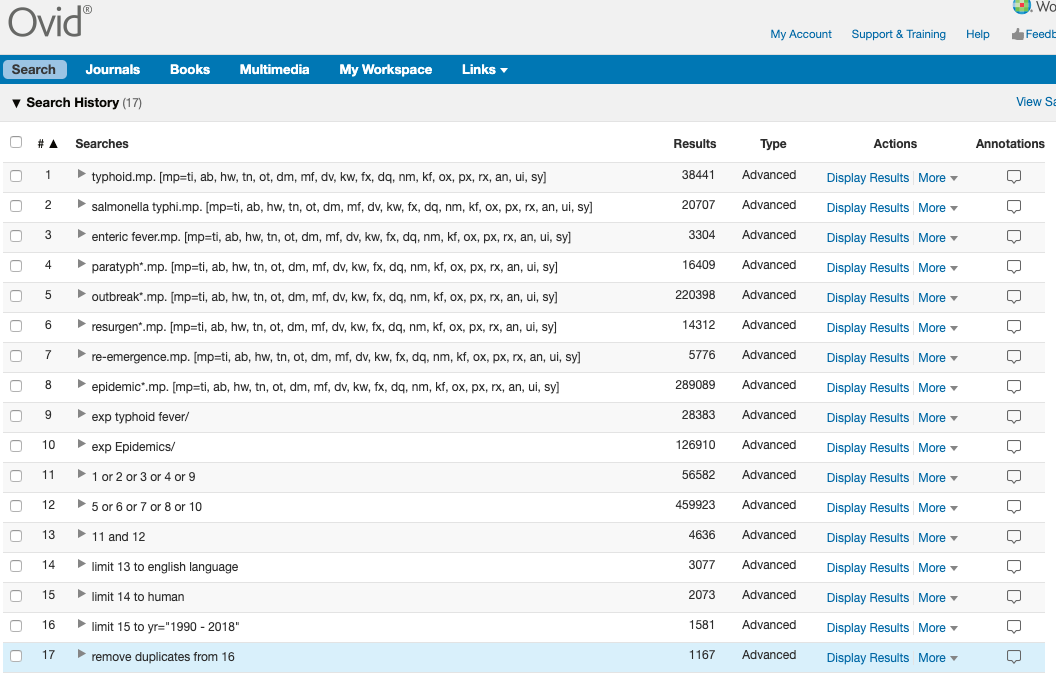


Figure 1: PRISMA Flow chart for the literature review of enteric fever outbreaks reported from 1 January 1990 to 31^st^ December 2016

Records identified through “Ovid MEDLINE (R) ALL” and “EMBASE Classic + EMBASE” using the Ovid interface

database (n=1581)

## Screening

## Included

## Eligibility

Records after removal of duplication (n=1167)

1

Records screened (n =2821)

Records excluded

(n =623)

Full-text articles assessed for eligibility (n=2198)

Full-text records excluded, with reasons (n=1909)

Studies included in quantitative synthesis
(n = 289 containing data for 303 unique outbreaks)

Records identified through ProMEDmail (n=1427)

Records identified through GIDEON online (n=227)

## Identification

| No. | Location (GPS co-ordinates) | Continent, country | Size of outbreak (n) | Case fatality rates (n) | Typhoid or paratyphoid | Month and year of outbreak | Year of Study | Source | S.typhi confirmation method | Multi-drug resistance | Putative cause | Comment if any |
| --- | --- | --- | --- | --- | --- | --- | --- | --- | --- | --- | --- | --- |
|  |  |  |  |  |  |  |  |  |  |  |  |  |
|  |  |  |  |  |  |  |  |  |  |  |  |  |

Step 14: Summarize the data as per below table in excel sheet (Data extraction table):

**Annex 2: Number of outbreaks and total number of reported enteric fever cases by region and sub-region for Asia**

| **Number** | **Location** | **(GPS co-ordinates)** | **Continent** | **Sub-region** | **Country** | **Size of outbreak (n)** | **Typhoid or Paratyphoid** | **Month and year of outbreak** | **Year** | **Confirmation method** | **MDR** | **Putative cause** |
| --- | --- | --- | --- | --- | --- | --- | --- | --- | --- | --- | --- | --- |
| 1 | Ontario, Canada | 51.194418, -86.507154 | North America |  | Canada | 11 | Typhoid | Feb-90, Mar-90 | 1990 | culture | unknown | foodborne |
| 2 | Gharbeya, Egypt | 30.854195, 31.049131 | Africa |  | Egypt | 133 | Typhoid | Nov-90, Dec-90 | 1990 | culture | yes | foodborne |
| 3 | Chandigarh, India | 30.724962, 76.780256 | Asia | Southern Asia | India | 62 | Typhoid | Apr-90, Jul-90 | 1990 | culture | yes | unknown |
| 4 | Calcutta, India | 22.598706, 88.396994 | Asia | Southern Asia | India | 40 | Typhoid | Jan-90 | 1990 | unknown | yes |  |
| 5 | Bangalore, India | 12.969871, 77.594162 | Asia | Southern Asia | India | 15 | Typhoid | Sep-90, Oct-90 | 1990 | culture | yes | unknown |
| 6 | Nagpur, India | 21.151320, 79.087273 | Asia | Southern Asia | India | 375 | Typhoid | Jul-90, Jun-91 | 1990 | culture | yes | unknown |
| 7 | New Delhi, India | 28.636506, 77.211334 | Asia | Southern Asia | India | 214 | Typhoid | Jan-90, Oct-90 | 1990 | culture | yes | unknown |
| 8 | Mumbai, India | 19.088091, 72.878458 | Asia | Southern Asia | India | 215 | Typhoid | Mar-90, | 1990 | culture | yes | unknown |
| 9 | Westmoreland, Jamaica | 18.228546, -78.056655 | North America |  | Jamaica | 321 | Typhoid | Oct-90, | 1990 | culture | unknown | unknown |
| 10 | Westmoreland, Jamaica | 18.228546, -78.056655 | North America |  | Jamaica | 154 | Typhoid | Aug-90, | 1990 | culture | unknown | contaminated water |
| 11 | Johor Bahru, Malaysia | 1.517398, 103.715118 | Asia | Southeastern Asia | Malaysia | 20 | Typhoid | Apr-90 | 1990 | culture | no |  |
| 12 | Singapore | 1.321238, 103.846051 | Asia | Southeastern Asia | Singapore | 95 | Typhoid | Apr-90, Sep-90 | 1990 | culture | no | person-person |
| 13 | Skagit County | 48.461211, -121.679295 | North America |  | USA | 18 | Typhoid | Jun-90 | 1990 | culture | no | foodborne |
| 14 | New York, USA | 40.726733, -73.998907 | North America |  | USA | 7 | Typhoid | Oct-90, Nov-90 | 1990 | culture | unknown | foodborne |
| 15 | Maryland, USA | 39.111146, -77.174560 | North America |  | USA | 24 | Typhoid | Aug-90 | 1990 | culture | unknown | foodborne |
| 16 | Massachusetts, USA | 42.392911, -71.736734 | North America |  | USA | 4 | Typhoid | Mar-90 | 1990 | culture | unknown | foodborne |
| 17 | Arizona, USA | 34.193150, -111.625822 | North America |  | USA | 21 | Typhoid | May-90 | 1990 | culture | unknown | foodborne |
| 18 | Porur, India | 13.041345, 80.156588 | Asia | Southern Asia | India | 236 | Typhoid | Jan-91 | 1991 | culture | yes | unknown |
| 19 | Duliajan, India | 27.356253, 95.322833 | Asia | Southern Asia | India | 286 | Paratyphoid + Typhoid | Jun-91, Mar-92 | 1991 | culture | yes | unknown |
| 20 | Pondicherry, India | 11.922786, 79.819342 | Asia | Southern Asia | India | 129 | Typhoid | 1991 | 1991 | unknown | yes | unknown |
| 21 | Bangalore, India | 12.969871, 77.594162 | Asia | Southern Asia | India | 190 | Typhoid | 1991 | 1991 | culture | yes | waterborne |
| 22 | Bangalore, India | 12.969871, 77.594162 | Asia | Southern Asia | India | 164 | Typhoid | Jun-91, May-92 | 1991 | culture | yes | unknown |
| 23 | Kota Bahru, Malaysia | 6.111947, 102.282578 | Asia | Southeastern Asia | Malaysia | 48 | Typhoid | Oct-91 | 1991 | unknown | no | unknown |
| 24 | Northern Natal, South Africa | 29.103401, 30.882236 | Africa |  | South Africa | 6 | Typhoid | Apr-91, Jun-91 | 1991 | culture | yes | contaminated water |
| 25 | Mostoles, Spain | 40.321667, -3.868695 | Europe |  | Spain | 54 | Typhoid | Jun-91, Jul-91 | 1991 | culture | no | foodborne |
| 26 | Port Vila Vanuatu | -17.735881, 168.325732 | Oceania |  | Vanuatu | 36 | Typhoid | Apr-91, | 1991 | culture | unknown | foodborne |
| 27 | Jajce, Bosnia and Herzegovina | 44.339372, 17.271161 | Europe |  | Bosnia and Herzegovnia | 16 | Typhoid | Nov-92, | 1992 | culture | no | contaminated water |
| 28 | Vidosi, Bosnia and Herzegovina | 43.771052, 17.028455 | Europe |  | Bosnia and Herzegovnia | 11 | Typhoid | Nov-92, | 1992 | culture | no | contaminated water |
| 29 | Zagreb, Yugoslavia | 45.805064, 15.979550 | Europe |  | Croatia | 25 | Typhoid | Nov-92 | 1992 | culture | unknown | contaminated water |
| 30 | Samoa | -13.671138, -172.461085 | Oceania |  | Samoa | 387 | Typhoid | 1992-1996 | 1992 | unknown | unknown | unknown |
| 31 | Al-Midhnab, Saudia Arabia | 25.862701, 44.227959 | Asia | Western Asia | Saudi Arabia | 19 | Typhoid | Jun-92, | 1992 | unknown | unknown | foodborne |
| 32 | Tabuk, Saudi Arabia | 28.389336, 36.568992 | Asia | Western Asia | Saudi Arabia | 185 | Typhoid | May-92 | 1992 | culture | no | contaminated water |
| 33 | East London | 51.516893, -0.043234 | Europe |  | UK | 5 | Typhoid | Jun-92 | 1992 | culture | unknown | foodborne |
| 34 | South West France | 44.514480, 0.925317 | Europe |  | France | 95 | Paratyphoid | Sep-93 | 1993 | unknown | unknown | foodborne |
| 35 | Shima, Japan | 34.326504, 136.835553 | Asia | Eastern Asia | Japan | 27 | Paratyphoid | Dec-93, Jan-94 | 1993 | culture | unknown | foodborne |
| 36 | Manila, Philippines | 14.597407, 120.984114 | Asia | Southeastern Asia | Philippeans | 252 | Typhoid | Jul-93, Apr-94 | 1993 | culture | yes | foodborne |
| 37 | Delmas, South Africa | -26.156040, 28.678143 | Africa |  | South Africa | 2129 | Typhoid | 1993 | 1993 | unknown | unknown | unknown |
| 38 | London, UK | 51.516893, -0.043234 | Europe |  | UK | 5 | Typhoid | Dec-90, | 1993 | culture | unknown | foodborne |
| 39 | Florida, USA | 27.541421, -81.509886 | North America |  | USA | 18 | Typhoid | Sep-93 | 1993 | culture | unknown | unknown |
| 40 | Kien Giang, Vietnam | 10.127538, 105.111913 | Asia | Southeastern Asia | Vietnam | 3049 | Typhoid | Apr-93, May-93 | 1993 | unknown | yes | unknown |
| 41 | Dhaka, Bangladesh | 23.824605, 90.414639 | Asia | Southern Asia | Bangladesh | 240 | Typhoid | 1994 | 1994 | culture | unknown | unknown |
| 42 | Lasva, Bosnia | 44.194309, 17.681085 | Europe |  | Bosnia and Herzegovnia | 28 | Typhoid | Mar-94 | 1994 | culture | no | contamined water |
| 43 | Zurich, Switzerland | 47.373016, 8.540833 | Europe |  | Switzerland | 10 | Typhoid | Apr-94 | 1994 | culture | unknown | foodborne |
| 44 | Thu Thiem, Vietnam | 10.772824, 106.715564 | Asia | Southeastern Asia | Vietnam | 364 | Typhoid | Mar-94, Jun-94 | 1994 | unknown | yes | unknown |
| 45 | Katkalamba, India | 18.853597, 77.363453 | Asia | Southern Asia | India | 415 | Typhoid | Nov-95, Dec-95 | 1995 | culture | unknown | contaminated water |
| 46 | Yavatmal, India | 20.387070, 78.118887 | Asia | Southern Asia | India | 33 | Paratyphoid | May-95, | 1995 | culture | unknown | foodborne |
| 47 | Tehran, Iran | 35.705457, 51.384838 | Asia | Southern Asia | Iran | 1121 | Typhoid | Jul-95 | 1995 | culture | yes | waterborne |
| 48 | Ain Taya, Algeria | 36.786507, 3.283966 | Africa |  | Algeria | 910 | Typhoid | Jan-96, | 1996 | culture | unknown | contaminated water |
| 49 | Northern Division, Fiji | -16.549753, 179.350354 | Oceania |  | Fiji | 25 | Typhoid | 1996 | 1996 | unknown | unknown | unknown |
| 50 | France | 46.993166, 2.472498 | Europe |  | France | 277 | Paratyphoid | Jun-96 | 1996 | unknown | no | unknown |
| 51 | New Delhi, India | 28.597825, 77.207487 | Asia | Southern Asia | India | 36 | Paratyphoid | Sep-96, Oct-96 | 1996 | culture | no | waterborne |
| 52 | Jeonbuk, Korea | 35.866817, 127.117285 | Asia | Eastern Asia | Korea | 232 | Typhoid | Aug-96, Oct-96 | 1996 | culture | yes | unknown |
| 53 | Busan, Korea | 35.147889, 129.040214 | Asia | Eastern Asia | Korea | 93 | Typhoid | Apr-96, Jun-96 | 1996 | culture | yes | contaminated water |
| 54 | Singapore | 1.315760, 103.826413 | Asia | Southeastern Asia | Singapore | 167 | Paratyphoid | Feb-96, May-96 | 1996 | culture | unknown | foodborne |
| 55 | Taiwan | 23.623597, 121.043075 | Asia | Eastern Asia | Taiwan | 5 | Paratyphoid | Oct-96 | 1996 | unknown | unknown | imported |
| 56 | Dushanbe, Tajikistan | 38.573404, 68.780894 | Asia | Central Asia | Tajikistan | 10677 | Typhoid | Jan-96, Jun-97 | 1996 | culture | yes | contaminated water |
| 57 | Kulyab, Tajikistan | 37.932311, 69.800777 | Asia | Central Asia | Tajikistan | 7516 | Typhoid | May-96 | 1996 | clinical | unknown | contaminated water |
| 58 | Bangkok, Thailand | 13.730864 | Asia | Southeastern Asia | Thailand | 9 | Paratyphoid | 1996 | 1996 | culture | no | foodborne |
| 59 | Florida, USA | 27.541421, -81.509887 | North America |  | USA | 9 | Typhoid | Nov-96 | 1996 | culture | unknown | foodborne |
| 60 | Juan Dolio, Dominican Republic | 18.443052, -69.410892 | North America |  | Dominican Republic | 4 | Typhoid | Jul-97 | 1997 | unknown | unknown | unknown |
| 61 | Utelle, France | 43.919865, 7.251313 | Europe |  | France | 26 | Typhoid | Aug-97 | 1997 | culture | no | foodborne |
| 62 | Ashkabad, Turkmenistan | 37.945341, 58.361521 | Asia | Central Asia | Turkmenistan | 300 | Typhoid | Mar-97 | 1997 | unknown | unknown | unknown |
| 63 | Glamorgan, Wales | 51.442837, -3.421642 | Europe |  | UK | 1 | Typhoid | Mar-97 | 1997 | unknown | unknown | foodborne |
| 64 | Armenia | 40.351681, 44.639740 | Asia | Western Asia | Armenia | 10 | Typhoid | 1998 | 1998 | unknown | unknown | unknown |
| 65 | Seine River, France | 48.850388, 2.358733 | Europe |  | France | 27 | Typhoid | Apr-98 | 1998 | culture | no | foodborne |
| 66 | Nauru | -0.528200, 166.934925 | Oceania |  | Nauru | 50 | Typhoid | Oct-98, May-99 | 1998 | culture | no | foodborne |
| 67 | Florida, USA | 25.760199, -80.352943 | North America |  | USA | 16 | Typhoid | Nov-98, Feb-99 | 1998 | culture | no | foodborne |
| 68 | Vanadzor, Armenia | 40.810097, 44.496045 | Asia | Western Asia | Armenia | 6 | Typhoid | Apr-99 | 1999 | unknown | unknown | contaminated water |
| 69 | Austrailia | -24.171230, 135.269350 | Oceania |  | Australia | 4 | Typhoid | May-99 | 1999 | unknown | unknown | foodborne |
| 70 | Xing-An County, China | 24.715468, 110.463341 | Asia | Eastern Asia | China | 24 | Typhoid | Ma-99, Jun-99 | 1999 | culture | unknown | foodborne |
| 71 | Zugdidi, Georgia | 42.512440, 41.865678 | Asia | Western Asia | Georgia | 24 | Typhoid | Apr-99 | 1999 | unknown | unknown | contaminated water |
| 72 | Guatemala city, Guatemala | 14.630519, -90.507546 | Central America |  | Guatemala | 120 | Typhoid | Mar-99 | 1999 | unknown | unknown | contaminated water |
| 73 | Izabal, Guatemala | 15.569206, -89.027457 | Central America |  | Guatemala | 24 | Typhoid | Apr-99 | 1999 | unknown | unknown | contaminated water |
| 74 | Mangalore, India | 12.908436, 74.855179 | Asia | Southern Asia | India | 16 | Typhoid | Sep-99, | 1999 | unknown | yes | contaminated water |
| 75 | Kottayam, India | 9.594033, 76.524884 | Asia | Southern Asia | India | 87 | Typhoid | Aug-99, Oct-99 | 1999 | unknown | unknown | unknown |
| 76 | Tokyo, Japan | 35.705131, 139.732361 | Asia | Eastern Asia | Japan | 7 | Typhoid | Mar-99 | 1999 | unknown | unknown | imported |
| 77 | Kavieng town, Papa New Guinea | -2.576305, 150.806505 | Oceania |  | Papua New Guinea | 600 | Typhoid | Jan-99 | 1999 | unknown | unknown | unknown |
| 78 | Kokoda Track, Papua New Guinea | -8.978669, 147.732682 | Oceania |  | Papua New Guinea | 19 | Typhoid | Jun-99 | 1999 | culture | unknown | unknown |
| 79 | Krasnoyarsk, Russia | 56.024043, 92.899380 | Europe |  | Russia | 13 | Typhoid | Mar-99 | 1999 | unknown | unknown | imported |
| 80 | Poppra District, Thailand | 16.495341, 98.861863 | Asia | Southeastern Asia | Thailand | 11 | Typhoid | Nov-99 | 1999 | culture | yes | contaminated water |
| 81 | Nuku'alofa, Tonga | -21.138570, -175.204811 | Oceania |  | Tonga | 9 | Typhoid | Apr-99 | 1999 | unknown | unknown | unknown |
| 82 | Alanya, Turkey (imported) | 36.546079, 31.999777 | Asia | Western Asia | Turkey | 309 | Paratyphoid | Aug-99, Sep-99 | 1999 | culture | unknown | contaminated water/ imported |
| 83 | Putumayo, Colombia | 0.624711, -75.986120 | South America |  | Colombia | 6 | Typhoid | 2000 | 2000 | culture | no | unknown |
| 84 | Mumbai, India | 19.088091, 72.878458 | Asia | Southern Asia | India | 150 | Typhoid | Nov-00 | 2000 | culture | yes | waterborne |
| 85 | Almaty, Khazakstan | 43.223228, 76.890607 | Asia | Central Asia | Khazakstan | 23 | Typhoid | Nov-00 | 2000 | unknown | unknown | foodborne |
| 86 | Hammana, Lebanon | 33.825276, 35.733518 | Asia | Western Asia | Lebanon | 27 | Typhoid | Mar-00 | 2000 | unknown | unknown | contaminated water |
| 87 | Madaya, Myanmar | 22.211759, 96.117840 | Asia | Southeastern Asia | Myanmar | 49 | Typhoid | Sep-00 | 2000 | unknown | no | contaminated water |
| 88 | Shakhtyorsk, Russia | 49.165575, 142.090240 | Europe |  | Russia | 7 | Typhoid | May-00 | 2000 | unknown | unknown | contaminated water |
| 89 | Yasnogorsk, Russia | 54.482298, 37.695297 | Europe |  | Russia | 19 | Typhoid | Oct-00 | 2000 | confirmed unknown | unknown | contaminated water |
| 90 | Upolu, Samoa | -13.913689, -171.735465 | Oceania |  | Samoa | 122 | Typhoid | Jan-00 | 2000 | culture | unknown | foodborne |
| 91 | Jizan, Saudi Arabia | 16.893814, 42.571571 | Asia | Western Asia | Saudi Arabia | 118 | Typhoid | Dec-00 | 2000 | unknown | unknown | unknown |
| 92 | Ohio- Kentucky-Indiana, USA | 39.106460, -84.661367 | North America |  | USA | 9 | Typhoid | May-00, Jul-00 | 2000 | culture | no | sex |
| 93 | Queens NY, USA | 40.738745, -73.799432 | North America |  | USA | 7 | Typhoid | Sept-00, | 2000 | culture | no | foodborne |
| 94 | Ivory Coast | 6.65295, -4.97589 | Africa |  | Ivory Coast | 24 | Typhoid | Aug-01, | 2001 | culture | no | foodborne |
| 95 | Thika, Kenya | -1.036688, 37.085037 | Africa |  | Kenya | 3 | Typhoid | Jan-01, Dec-04 | 2001 | culture | yes | unknown |
| 96 | Nairobi, Kenya | -1.299785, 36.808492 | Africa |  | Kenya | 85 | Typhoid | Jan-01, Dec-02 | 2001 | unknown | yes | unknown |
| 97 | Embu, Kenya | -0.537850, 37.458865 | Africa |  | Kenya | 100 | Typhoid | Feb-01 | 2001 | culture | yes | contaminated water |
| 98 | Nakuru, Kenya | -0.293496, 36.083664 | Africa |  | Kenya | 189 | Typhoid | Jun-01 | 2001 | unknown | unknown | contaminated water |
| 99 | Batken, Kyrgyzstan | 39.721754, 70.715180 | Asia | Central Asia | Kyrgyzstan | 4 | Typhoid | Aug-01 | 2001 | unknown | unknown | unknown |
| 100 | Kathmandu, Nepal | 27.721042, 85.324760 | Asia | Southern Asia | Nepal | 74 | Typhoid | Dec-01, May-02 | 2001 | culture | unknown | unknown |
| 101 | Gwent, UK | 51.665262, -2.987979 | Europe |  | UK | 3 | Typhoid | 2001, | 2001 | unknown | unknown | unknown |
| 102 | Newport, Wales | 51.581555, -2.998165 | Europe |  | UK | 5 | Typhoid | Jul-01 | 2001 | culture | unknown | foodborne |
| 103 | Antioquia, Colombia | 6.691446, -75.515138 | South America |  | Colombia | 4 | Typhoid | 2002 | 2002 | culture | no | unknown |
| 104 | Mombasa, Kenya | -4.048542, 39.658292 | Africa |  | Kenya | 39 | Typhoid | May-02 | 2002 | unknown | unknown | contaminated water |
| 105 | Busan, Korea | 35.147889, 129.040214 | Asia | Eastern Asia | Korea | 200 | Paratyphoid | 2002 (early) | 2002 | culture | yes | contaminated water |
| 106 | Bharatpur, Nepal | 27.682300, 84.437466 | Asia | Southern Asia | Nepal | 5963 | Typhoid | May-02. Jul-02 | 2002 | culture | yes | contaminated water |
| 107 | Bambang, Philippeans | 16.387622, 121.111095 | Asia | Southeastern Asia | Philippeans | 26 | Typhoid | Jul-02 | 2002 | unknown | unknown | contaminated water |
| 108 | Samoa | -13.865489, -171.935853 | Oceania |  | Samoa | 200 | Typhoid | May-02 | 2002 | unknown | unknown | unknown |
| 109 | Taipei, Taiwan | 25.040440, 121.518958 | Asia | Eastern Asia | Taiwan | 24 | Typhoid | Jun-02, | 2002 | culture | no | contaminated water |
| 110 | Murghob, Tajikistan | 38.185710, 73.998113 | Asia | Central Asia | Tajikistan | 51 | Typhoid | Dec-02 | 2002 | confirmed unknown | unknown | contaminated water |
| 111 | Thuan Chau, Vietnam | 21.429250, 103.618927 | Asia | Southeastern Asia | Vietnam | 28 | Typhoid | Sep-02, Oct-02 | 2002 | culture | no | contaminated water |
| 112 | Quynh Nhai, Vietnam | 21.775943, 103.650341 | Asia | Southeastern Asia | Vietnam | 23 | Typhoid | Jul-02, Aug-02 | 2002 | culture | no | contaminated water |
| 113 | Phu Yen, Vietnam | 21.264430, 104.643906 | Asia | Southeastern Asia | Vietnam | 32 | Typhoid | Nov-02, | 2002 | culture | no | contaminated water |
| 114 | Megri, Armenia | 38.904064, 46.246841 | Asia | Western Asia | Armenia | 20 | Typhoid | Sep-03 | 2003 | unknown | unknown | contaminated water |
| 115 | Wenzhou, China | 27.998069, 120.698008 | Asia | Eastern Asia | China | 85 | Paratyphoid | Jan-03 | 2003 | culture | no | contaminated water |
| 116 | Paris, France | 48.859537, 2.351180 | Europe |  | France | 5 | Typhoid | Nov-03 | 2003 | unknown | unknown | foodborne |
| 117 | Ashanti, Ghana | 6.814074, -1.512845 | Africa |  | Ghana | 20 | Typhoid | Dec-03 | 2003 | unknown | unknown | contaminated water |
| 118 | Grand Bois, Haiti | 19.350080, -71.951319 | North America |  | Haiti | 200 | Typhoid | 2003 | 2003 | unknown | unknown | unknown |
| 119 | Maharastra, India | 19.362153, 75.521729 | Asia | Southern Asia | India | 95 | Typhoid | Mar-03 | 2003 | culture | yes | waterborne |
| 120 | Tehran, Iran | 35.705457, 51.384838 | Asia | Southern Asia | Iran | 535 | Paratyphoid | Jul-03, | 2003 | culture | yes | unknown |
| 121 | Batken, Kyrgyzstan | 39.721754, 70.715180 | Asia | Central Asia | Kyrgyzstan | 200 | Typhoid | Oct-03 | 2003 | unknown | unknown | contaminated water |
| 122 | Bacoor, Philippines | 14.409209, 120.975520 | Asia | Southeastern Asia | Philippeans | 37 | Typhoid | Dec-03 | 2003 | unknown | unknown | foodborne |
| 123 | Dushanbe, Tajikistan | 38.573404, 68.780893 | Asia | Central Asia | Tajikistan | 1000 | Typhoid | Oct-03 | 2003 | unknown | unknown | contaminated water |
| 124 | Batna, Algeria | 35.554988, 6.170143 | Africa |  | Algeria | 328 | Typhoid | Sep-04 | 2004 | unknown | unknown | contaminated water |
| 125 | Buenos Aires, Argentina | -34.623302, -58.434233 | South America |  | Argentina | 3 | Typhoid | 2004 | 2004 | culture | no | unknown |
| 126 | Dhaka, Bangladesh | 23.796658, 90.414519 | Asia | Southern Asia | Bangladesh | 8 | Typhoid | Mar-04, Apr-04 | 2004 | culture | yes | unknown |
| 127 | Guangxi, China | 23.547017, 109.023233 | Asia | Eastern Asia | China | 394 | Paratyphoid | Nov-04 | 2004 | culture | unknown | waterborne |
| 128 | Famagusta, Cyprus | 35.116912, 33.921833 | Asia | Western Asia | Cyprus | 20 | Typhoid | Aug-04 | 2004 | unknown | unknown | unknown |
| 129 | Kinshasa, Democratic Republic of Congo | -4.309123, 15.305952 | Africa |  | Democratic Republic of Congo | 13400 | Typhoid | Oct-04 | 2004 | culture | yes | waterborne |
| 130 | Westmoreland, Jamaica | 18.241916, -78.064208 | North America |  | Jamaica | 5 | Typhoid | Feb-04 | 2004 | unknown | unknown | unknown |
| 131 | Jordan Valley, Jordan | 32.204545, 35.611756 | Asia | Western Asia | Jordan | 83 | Typhoid | Oct-04, Jan-05 | 2004 | culture | yes | unknown |
| 132 | Batken, Kyrgyzstan | 39.721754, 70.715180 | Asia | Central Asia | Kyrgyzstan | 110 | Typhoid | May-04 | 2004 | confirmed unknown | unknown | contaminated water |
| 133 | Jalal-Abad, Kyrgyzstan | 40.935715, 72.980890 | Asia | Central Asia | Kyrgyzstan | 63 | Typhoid | Oct-04 | 2004 | confirmed unknown | unknown | contaminated water |
| 134 | Osh City, Kyrgzstan | 40.519428, 72.814035 | Asia | Central Asia | Kyrgyzstan | 103 | Typhoid | 2004 | 2004 | confirmed unknown | unknown | unknown |
| 135 | Talas, Kyrgyzstan | 42.529424, 72.228756 | Asia | Central Asia | Kyrgyzstan | 71 | Typhoid | Aug-04 | 2004 | confirmed unknown | unknown | unknown |
| 136 | Nek Muhammad, Pakistan | 27.985747, 69.331200 | Asia | Southern Asia | Pakistan | 300 | Typhoid | Oct-04, | 2004 | culture | yes | contaminated water |
| 137 | Morobe, Papa New Guinea | -7.770852, 147.593523 | Oceania |  | Papua New Guinea | 160 | Typhoid | Mar-04 | 2004 | unknown | unknown | unknown |
| 138 | Komi, Russia | 64.631250, 55.564817 | Europe |  | Russia | 1 | Typhoid | Mar-04 | 2004 | unknown | unknown | foodborne |
| 139 | Moscow, Russia | 55.748040, 37.623925 | Europe |  | Russia | 27 | Typhoid | Jul-04 | 2004 | culture | unknown | contaminated water |
| 140 | Ruhengeri, Rwanda | -1.502232, 29.633184 | Africa |  | Rwanda | 540 | Typhoid | Jul-04 | 2004 | unknown | unknown | contaminated water |
| 141 | Jaffna, Sri Lanka | 9.662372, 80.025860 | Asia | Southern Asia | Sri Lanka | 300 | Typhoid | Jul-04 | 2004 | unknown | unknown | contaminated water |
| 142 | Dushanbe, Tajikistan | 38.573404, 68.780891 | Asia | Central Asia | Tajikistan | 100 | Typhoid | Jul-04 | 2004 | unknown | yes | contaminated water |
| 143 | Hatlonskaya, Tajikistan | 37.838170, 68.781284 | Asia | Central Asia | Tajikistan | 79 | Typhoid | Jun-04 | 2004 | unknown | unknown | contaminated water |
| 144 | Kolkhozabad, Tajikistan | 37.603131, 68.605027 | Asia | Central Asia | Tajikistan | 50 | Typhoid | Dec-04 | 2004 | unknown | unknown | contaminated water |
| 145 | Shahrinav, Tajikistan | 38.572368, 68.336163 | Asia | Central Asia | Tajikistan | 34 | Typhoid | Jun-04 | 2004 | unknown | unknown | contaminated water |
| 146 | Gabes, Tunisia | 33.887177, 10.102916 | Africa |  | Tunisia | 39 | Typhoid | Nov-04, | 2004 | culture | no | foodborne |
| 147 | Odessa, Ukraine | 46.479567, 30.723491 | Europe |  | Ukraine | 11 | Typhoid | Jul-04 | 2004 | unknown | unknown | foodborne |
| 148 | Mekong Delta, Vietnam | 10.120665, 105.926894 | Asia | Southeastern Asia | Vietnam | 267 | Typhoid | Jan-04 | 2004 | culture | no | unknown |
| 149 | El-Oued, Algeria | 33.369150, 6.856313 | Africa |  | Algeria | 49 | Typhoid | May-Sep 2005 | 2005 | culture | unknown | waterborne |
| 150 | Central/ Eastern Divisions, Fiji | -14.571428, 28.312030 | Oceania |  | Fiji | 100 | Typhoid | Jun-05 | 2005 | unknown | unknown | unknown |
| 151 | Wainunu, Fiji | -16.932558, 178.886740 | Oceania |  | Fiji | 45 | Typhoid | Aug-05 | 2005 | unknown | unknown | unknown |
| 152 | French Guiana | 4.180063, -53.027149 | South America |  | French Guiana | 13 | Typhoid | 2005 | 2005 | unknown | unknown | unknown |
| 153 | Oyem, Gabon | 1.598175, 11.576028 | Africa |  | Gabon | 100 | Typhoid | Jan-05 | 2005 | unknown | unknown | contaminated water |
| 154 | Yuen Long District, Hong Kong | 22.464469, 114.058900 | Asia | Eastern Asia | Hong Kong | 19 | Typhoid | Nov-05 | 2005 | unknown | unknown | unknown |
| 155 | Central India | 22.395648, 79.223332 | Asia | Southern Asia | India | 98 | Typhoid | Aug-05 | 2005 | culture | unknown | waterborne |
| 156 | Bungoma, Kenya | 0.571558, 34.557495 | Africa |  | Kenya | 150 | Typhoid | Mar-05 | 2005 | unknown | unknown | contaminated water |
| 157 | Kara-Suu, Kyrgyzstan | 40.709693, 72.879492 | Asia | Central Asia | Kyrgyzstan | 14 | Typhoid | Jun-05 | 2005 | unknown | unknown | unknown |
| 158 | Kyrgyzstan | 41.375500, 74.573984 | Asia | Central Asia | Kyrgyzstan | 78 | Typhoid | Sep-05 | 2005 | unknown | unknown | unknown |
| 159 | Kelantan, Malaysia | 6.085139, 102.244393 | Asia | Southeastern Asia | Malaysia | 735 | Typhoid | Apr-05 | 2005 | culture | unknown | unknown |
| 160 | Borbon, Philippines | 10.842222, 123.997419 | Asia | Southeastern Asia | Philippeans | 250 | Typhoid | Oct-05 | 2005 | unknown | unknown | unknown |
| 161 | Dagupenos, Philippines | 16.039089, 120.335579 | Asia | Southeastern Asia | Philippeans | 5 | Typhoid | Sep-05 | 2005 | unknown | unknown | contaminated water |
| 162 | Pasay, Philippines | 14.535536, 121.001618 | Asia | Southeastern Asia | Philippeans | 39 | Typhoid | Jan-05 | 2005 | unknown | unknown | contaminated water |
| 163 | St. Petersburg, Russia | 59.942346, 30.331353 | Europe |  | Russia | 49 | Typhoid | Dec-05 | 2005 | unknown | unknown | unknown |
| 164 | Delmas, South Africa | -26.156040, 28.678143 | Africa |  | South Africa | 600 | Typhoid | Sep-05-Dec-05 | 2005 | culture | no | contaminated water |
| 165 | Nongoma, South Africa | -27.895868, 31.647292 | Africa |  | South Africa | 5 | Typhoid | Sep-05 | 2005 | unknown | unknown | contaminated water |
| 166 | Transkei, South Africa | -31.631617, 28.857253 | Africa |  | South Africa | 15 | Typhoid | May-05 | 2005 | unknown | unknown | unknown |
| 167 | Taoyuan, Taiwan | 24.989471, 121.297283 | Asia | Eastern Asia | Taiwan | 14 | Typhoid | Jun-05 | 2005 | culture | unknown | unknown |
| 168 | Zakarpatye, Ukraine | 48.340266, 23.376149 | Europe |  | Ukraine | 30 | Typhoid | Dec-05, | 2005 | confirmed unknown | unknown | contaminated water |
| 169 | New York, USA | 40.700189, -74.005774 | North America |  | USA | 2 | Typhoid | 2005 | 2005 | unknown | unknown | imported |
| 170 | Yunnan, China | 24.504619, 101.621151 | Asia | Eastern Asia | China | 39 | Paratyphoid + Typhoid | Aug-06 | 2006 | unknown | unknown | unknown |
| 171 | Northern Divison, Fiji | -16.549753, 179.350360 | Oceania |  | Fiji | 113 | Typhoid | Aug-06 | 2006 | unknown | unknown | unknown |
| 172 | Kerala, India | 9.443420, 76.750999 | Asia | Southern Asia | India | 82 | Typhoid | Aug-06 | 2006 | unknown | unknown | contaminated water |
| 173 | Jalal-Abad, Kyrgyzstan | 40.935715, 72.980890 | Asia | Central Asia | Kyrgyzstan | 5 | Typhoid | Feb-06 | 2006 | confirmed unknown | unknown | contaminated water |
| 174 | Lebanon | 34.180856, 35.907694 | Asia | Western Asia | Lebanon | 30 | Typhoid | Nov-06 | 2006 | unknown | unknown | unknown |
| 175 | Majuro, Marshall Islands | 7.110237, 171.185369 | Oceania |  | Marshall Islands | 22 | Typhoid | Mar-06 | 2006 | unknown | unknown | unknown |
| 176 | Manthali, Nepal | 27.386115, 86.071916 | Asia | Southern Asia | Nepal | 200 | Typhoid | Nov-06 | 2006 | unknown | unknown | contaminated water |
| 177 | Sankhuwasabha, Nepal | 27.669068, 87.227240 | Asia | Southern Asia | Nepal | 20 | Typhoid | Oct-06 | 2006 | unknown | unknown | unknown |
| 178 | Erave, Papa New Guinea | -6.327496, 143.840046 | Oceania |  | Papua New Guinea | 1200 | Typhoid | Feb-06 | 2006 | unknown | unknown | unknown |
| 179 | Isabela, Philippines | 17.041246, 121.978819 | Asia | Southeastern Asia | Philippeans | 100 | Typhoid | Jan-06 | 2006 | unknown | unknown | unknown |
| 180 | Mindanao, Philippines | 8.494560, 123.303674 | Asia | Southeastern Asia | Philippeans | 478 | Typhoid | Oct-06 | 2006 | unknown | unknown | contaminated water |
| 181 | St. Petersburg, Russia | 59.942346, 30.331353 | Europe |  | Russia | 67 | Typhoid | Sep-06 | 2006 | unknown | unknown | foodborne |
| 182 | Limpopo, South Africa | -23.974835, 29.541198 | Africa |  | South Africa | 12 | Typhoid | Feb-06 | 2006 | confirmed unknown | unknown | unknown |
| 183 | Charsada, Afghanistan | 34.477211, 65.258570 | Asia | Southern Asia | Afghanistan | 200 | Typhoid | Feb-07 | 2007 | unknown | unknown | unknown |
| 184 | Reguiba, Algeria | 33.569372, 6.716175 | Africa |  | Algeria | 61 | Typhoid | May-Sep 2007 | 2007 | culture | unknown | waterborne |
| 185 | Djelfa, Algeria | 34.662460, 3.339183 | Africa |  | Algeria | 36 | Typhoid | Aug-07 | 2007 | unknown | unknown | unknown |
| 186 | Jujuy, Argentina | -22.996381, -65.718144 | South America |  | Argentina | 15 | Typhoid | Jan-07 | 2007 | unknown | unknown | foodborne |
| 187 | Armenia | 40.395747, 44.606130 | Asia | Western Asia | Armenia | 5 | Paratyphoid | Sep-07 | 2007 | culture |  |  |
| 188 | Northern Divison, Fiji | -16.549753, 179.350358 | Oceania |  | Fiji | 95 | Typhoid | Mar-07 | 2007 | unknown | unknown | contaminated water |
| 189 | Northern Divison, Fiji | -16.549753, 179.350357 | Oceania |  | Fiji | 38 | Typhoid | Aug-07 | 2007 | unknown | unknown | unknown |
| 190 | South Dumdum, India | 22.604031, 88.399050 | Asia | Southern Asia | India | 103 | Typhoid | Feb-07,Apr-07 | 2007 | culture | unknown | contaminated water/ foodborne |
| 191 | Varkana village, India | 25.342043, 73.357102 | Asia | Southern Asia | India | 219 | Typhoid | May-07, Jul-07 | 2007 | WIDAL clinical | yes | contaminated water |
| 192 | Hyderabad, India | 17.393954, 78.475325 | Asia | Southern Asia | India | 6 | Typhoid | Jun-07 | 2007 | unknown |  | contaminated water |
| 193 | Manipur, India | 24.719061, 93.758282 | Asia | Southern Asia | India | 83 | Typhoid | Jun-07 | 2007 | unknown | unknown | unknown |
| 194 | Majuro, Marshall Islands | 7.110237, 171.185369 | Oceania |  | Marshall Islands | 24 | Typhoid | Mar-07 | 2007 | unknown | unknown | contaminated water |
| 195 | Baglung, Nepal | 28.341786, 83.298608 | Asia | Southern Asia | Nepal | 50 | Typhoid | Jun-07 | 2007 | unknown |  | contaminated water |
| 196 | Porirua, New Zealand | -41.109332, 174.872937 | Oceania |  | New Zealand | 3 | Typhoid | Jan-07 | 2007 | culture | unknown | foodborne |
| 197 | Manukau, New Zealand | -36.993391, 174.874893 | Oceania |  | New Zealand | 4 | Typhoid | Jan-07 | 2007 | unknown | unknown | foodborne |
| 198 | Islamabad, Pakistan | 33.681627, 73.003507 | Asia | Southern Asia | Pakistan | 120 | Typhoid | Jun-07 | 2007 | unknown |  | contaminated water |
| 199 | Daru, Papa New Guinea | -9.080236, 143.207946 | Oceania |  | Papua New Guinea | 59 | Typhoid | Nov-07 | 2007 | unknown | unknown | contaminated water |
| 200 | Enga, Papa New Guinea | -5.445933, 143.502262 | Oceania |  | Papua New Guinea | 400 | Typhoid | Sep-07 | 2007 | unknown | unknown | contaminated water |
| 201 | Caraga, Philippines | 7.354605, 126.442336 | Asia | Southeastern Asia | Philippeans | 13 | Typhoid | Jan-07 | 2007 | confirmed unknown | unknown | unknown |
| 202 | Catanduanes, Philippines | 13.765176, 124.252351 | Asia | Southeastern Asia | Philippeans | 115 | Typhoid | Dec-07 | 2007 |  |  |  |
| 203 | Mindanao, Philippines | 8.494560, 123.303674 | Asia | Southeastern Asia | Philippeans | 200 | Typhoid | Apr-07 | 2007 | culture | unknown | contaminated water |
| 204 | Moscow, Russia | 55.748040, 37.623925 | Europe |  | Russia | 3 | Typhoid | Jan-07 | 2007 | unknown | unknown | unknown |
| 205 | Mus, Turkey | 38.733190, 41.493419 | Asia | Western Asia | Turkey | 70 | Typhoid | Mar-07 | 2007 | unknown | unknown | contaminated water |
| 206 | Kasese, Uganda | 0.172434, 30.078159 | Africa |  | Uganda | 577 | Typhoid | Dec-07, Jul-09 | 2007 | culture | unknown | unknown |
| 207 | Hubei, China | 31.171354, 112.789101 | Asia | Eastern Asia | China | 81 | Typhoid | Dec-08 | 2008 | unknown | unknown | unknown |
| 208 | Northern Divison, Fiji | -16.549753, 179.350356 | Oceania |  | Fiji | 3 | Typhoid | Feb-08 | 2008 | unknown | unknown | unknown |
| 209 | Sulaymaniyah City, Iraq | 35.570100, 45.377655 | Asia | Western Asia | Iraq | 3010 | Typhoid | Aug-08, May-09 | 2008 | culture | unknown | unknown |
| 210 | Kelantan, Malaysia | 6.085139, 102.244393 | Asia | Southeastern Asia | Malaysia | 64 | Typhoid | Apr-08 | 2008 | culture | unknown | waterborne |
| 211 | Porirua, New Zealand | -41.109332, 174.872937 | Oceania |  | New Zealand | 6 | Typhoid | Dec-08 | 2008 | culture | unknown | foodborne |
| 212 | Milne Bay, Papa New Guinea | -10.324290, 150.272288 | Oceania |  | Papua New Guinea | 20 | Typhoid | Dec-08 | 2008 | confirmed unknown | unknown | unknown |
| 213 | Iloilo, Philippines | 10.716718, 122.562785 | Asia | Southeastern Asia | Philippeans | 217 | Typhoid | Jan-08 | 2008 | unknown | unknown | contaminated water |
| 214 | Kisulad, Philippines | 6.526641, 125.540766 | Asia | Southeastern Asia | Philippeans | 100 | Typhoid | Jan-08 | 2008 | unknown | unknown | contaminated water |
| 215 | Laguna, Philippines | 14.245937, 121.441108 | Asia | Southeastern Asia | Philippeans | 1800 | Typhoid | Feb-08 | 2008 | confirmed unknown | unknown | contaminated water |
| 216 | Nueva Vizcaya, Philippines | 16.309196, 121.127679 | Asia | Southeastern Asia | Philippeans | 103 | Typhoid | Jan-08 | 2008 | unknown | unknown | contaminated water |
| 217 | Quezon, Philippines | 14.101152, 121.783739 | Asia | Southeastern Asia | Philippeans | 100 | Typhoid | Nov-08 | 2008 | unknown | unknown | unknown |
| 218 | Ercek, Turkey | 40.410263, 27.795290 | Asia | Western Asia | Turkey | 867 | Typhoid | Oct-08 | 2008 | culture | yes | waterborne |
| 219 | Taveuni, Fiji | -16.844665, -179.985226 | Oceania |  | Fiji | 20 | Typhoid | Jul-09 | 2009 | confirmed unknown | unknown | person-person |
| 220 | Chandigarh, India | 30.729347, 76.778774 | Asia | Southern Asia | India | 27 | Typhoid | Dec-09 Jan-10 | 2009 | unknown | yes | unknown |
| 221 | Selangor, Malaysia | 3.208812, 101.843410 | Asia | Southeastern Asia | Malaysia | 12 | Typhoid | Jan-09, Feb-09 | 2009 | culture | unknown | contaminated water |
| 222 | Neno District, Malawi | -15.659156, 34.564445 | Africa |  | Malawi | 784 | Typhoid | Jun-09 | 2009 | culture | yes | unknown |
| 223 | Pokhara, Nepal | 28.233334, 83.988545 | Asia | Southern Asia | Nepal | 37 | Paratyphoid | Oct-09 | 2009 | culture | unknown | unknown |
| 224 | Songkhla, Thailand | 7.190197, 100.602735 | Asia | Southeastern Asia | Thailand | 137 | Typhoid | Oct-09, Mar-10 | 2009 | culture | no | unknown |
| 225 | Kasese, Uganda | 0.172434, 30.078159 | Africa |  | Uganda | 1341 | Typhoid | Aug-09, Dec-11 | 2009 | culture | yes | contaminated water |
| 226 | Internally displaced persons camp, Uganda | 1.323172, 32.299648 | Africa |  | Uganda | 81 | Typhoid | 2009-2013 | 2009 | culture | yes | unknown |
| 227 | Tennessee, USA | 35.571792, -86.670738 | North America |  | USA | 3 | Typhoid | Jun-09 | 2009 | confirmed unknown | unknown | imported |
| 228 | Yuanjiang, China | 28.992272, 112.551405 | Asia | Eastern Asia | China | 601 | Paratyphoid | May-10 | 2010 | culture | unknown | contaminated water |
| 229 | Shache, China | 38.386680, 76.979852  38.386680, 76.979852  38.386680, 76.979852  38.386680, 76.979852  38.386680, 76.979852  38.386680, 76.979852 | Asia | Eastern Asia | China | 253 | Typhoid | Jul-10 | 2010 | culture | yes | waterborne |
| 230 | GUANGXI ZHUANG, China | 23.374677, 108.813231 | Asia | Eastern Asia | China | 84 | Paratyphoid | Sep-10 | 2010 |  |  |  |
| 231 | Northern Divison, Fiji | -16.549753, 179.350359 | Oceania |  | Fiji | 112 | Typhoid | Mar-10 | 2010 | unknown | unknown | contaminated water |
| 232 | Andhra Pradesh, India | 14.814880, 79.046387 | Asia | Southern Asia | India | 40 | Paratyphoid | 2010 | 2010 | unknown | unknown | imported |
| 233 | Haryana, India | 29.004661, 76.290204 | Asia | Southern Asia | India | 43 | Typhoid | Aug-10 | 2010 | unknown | unknown | contaminated water |
| 234 | Russia (Far East) | 61.331176, 106.164063 | Europe |  | Russia | 40 | Typhoid | Jan-10 | 2010 | confirmed unknown | unknown | imported |
| 235 | Pretoria, South Africa | -25.755867, 28.210756 | Africa |  | South Africa | 10 | Typhoid | Apr-10, May-10 | 2010 | culture | no | foodborne |
| 236 | Hsinchu County, Taiwan | 24.680451, 121.165985 | Asia | Eastern Asia | Taiwan | 15 | Typhoid | Jan-10 | 2010 | culture | unknown | foodborne |
| 237 | South Nevada, USA | 35.941101, -115.041260 | North America |  | USA | 12 | Typhoid | Apr-10, Sep-10 | 2010 | culture | unknown | foodborne |
| 238 | Phnom Penh, Cambodia | 11.562883, 104.879887 | Asia | Southeastern Asia | Cambodia | 71 | Paratyphoid | Jan-11, Aug-13 | 2011 | culture | no | unknown |
| 239 | Lusaka, Zambia | -15.410393, 28.347241 | Africa |  | Zambia | 2040 | Typhoid | Dec-11 | 2011 | culture | yes | unknown |
| 240 | Mufulira, Zambia | -12.554013, 28.243489 | Africa |  | Zambia | 4396 | Typhoid | Dec-11 | 2011 | unknown | unknown | unknown |
| 241 | Harare, Zimbabwe | -17.823501, 31.060978 | Africa |  | Zimbabwe | 4181 | Typhoid | Oct-11, Apr-12 | 2011 | culture | no | contaminated water |
| 242 | Ba, Fiji | -17.531808, 177.664268 | Oceania |  | Fiji | 15 | Typhoid | Mar-12 | 2012 | unknown | unknown | unknown |
| 243 | Nanoko, Fiji | -17.755798, 177.841465 | Oceania |  | Fiji | 42 | Typhoid | Jan-12 | 2012 | confirmed unknown | unknown | contaminated water + person-person |
| 244 | Viti Levu, Fiji | -17.848355, 178.011870 | Oceania |  | Fiji | 28 | Typhoid | Mar-12 | 2012 | unknown | unknown | unknown |
| 245 | Himachal Pradesh, India | 31.097680, 77.184452 | Asia | Southern Asia | India | 15 | Typhoid | Feb-12 | 2012 | unknown | unknown | contaminated water |
| 246 | Karnataka, India | 14.549866, 75.746183 | Asia | Southern Asia | India | 360 | Typhoid | Feb-12 | 2012 | unknown | unknown | contaminated water |
| 247 | Japan | 35.705131, 139.732361 | Asia | Eastern Asia | Japan | 18 | Paratyphoid | Dec-12 | 2012 | culture | unknown | unknown |
| 248 | Philippines | 14.597407, 120.984114 | Asia | Southeastern Asia | Philippeans | 2 | Typhoid | May-12 | 2012 | culture | unknown | unknown |
| 249 | Alegria, Philippines | 9.762849, 123.375601 | Asia | Southeastern Asia | Philippeans | 11 | Typhoid | Mar-12 | 2012 | unknown | unknown | unknown |
| 250 | Leyte, Philippines | 10.862118, 124.881447 | Asia | Southeastern Asia | Philippeans | 102 | Typhoid | Jan-12 | 2012 | unknown | unknown | contaminated water |
| 251 | Tuburan, Philippines | 10.711627, 123.862272 | Asia | Southeastern Asia | Philippeans | 999 | Typhoid | Mar-12 | 2012 | confirmed unknown | unknown | unknown |
| 252 | Malie, Samoa | -13.908724, -171.752348 | Oceania |  | Samoa | 9 | Typhoid | Mar-12 | 2012 |  |  |  |
| 253 | Upolo, Samoa | -13.919548, -171.732389 | Oceania |  | Samoa | 10 | Typhoid | Apr-12 | 2012 | confirmed unknown | unknown | contaminated water |
| 254 | New Taipei City, Taiwan | 25.014446, 121.463852 | Asia | Eastern Asia | Taiwan | 1 | Typhoid | Apr-12 | 2012 | culture | unknown | foodborne |
| 255 | Tongatapu, Tonga | -21.150084, -175.248500 | Oceania |  | Tonga | 2 | Typhoid | Mar-12 | 2012 | unknown | unknown | contaminated water |
| 256 | Kabwe, Zambia | -14.424484, 28.452030 | Africa |  | Zambia | 204 | Typhoid | Apr-12 | 2012 | unknown | unknown | unknown |
| 257 | American Samoa | -14.262436, -170.663566 | Oceania |  | American Samoa | 8 | Typhoid | Jun-13 | 2013 | confirmed unknown | unknown | unknown |
| 258 | Phnom Penh, Cambodia | 11.562883, 104.879887 | Asia | Southeastern Asia | Cambodia | 75 | Paratyphoid | Jan-13 | 2013 | culture |  | contaminated water |
| 259 | Kasai Occidental, Democratic Republic of Congo | -5.514094, 21.788414 | Africa |  | Democratic Republic of Congo | 1092 | Typhoid | May-13 | 2013 | unknown | unknown | contaminated water |
| 260 | Taveuni, Fiji | -16.844665, -179.985226 | Oceania |  | Fiji | 7 | Typhoid | May-13 | 2013 | unknown | unknown | unknown |
| 261 | Gujarat, India | 23.105718, 70.927519 | Asia | Southern Asia | India | 25 | Typhoid | May-13 | 2013 | unknown | unknown | contaminated water |
| 262 | Wagoora, India | 34.173702, 74.432571 | Asia | Southern Asia | India | 300 | Typhoid | Jun-13 | 2013 | unknown | unknown | contaminated water |
| 263 | Blantyre, Malawi | -15.784457, 35.012223 | Africa |  | Malawi | 843 | Typhoid | 2013 | 2013 | culture | yes | unknown |
| 264 | Malawi | -13.771806, 34.053848 | Africa |  | Malawi | 30000 | Typhoid | Jan-13 | 2013 | culture | unknown | unknown |
| 265 | Kelantan, Malaysia | 6.085139, 102.244393 | Asia | Southeastern Asia | Malaysia | 10 | Typhoid | Mar-13 | 2013 | culture | unknown | unknown |
| 266 | Oas, Philippines | 13.169707, 123.415432 | Asia | Southeastern Asia | Philippeans | 66 | Typhoid | Jun-13 | 2013 | unknown | unknown | unknown |
| 267 | Dier Ezzor, Syria | 35.333577, 40.135229 | Asia | Western Asia | Syria | 1200 | Typhoid | 2013 | 2013 | unknown | unknown | unknown |
| 268 | Central Province, Zambia | -14.571428, 28.312030 | Africa |  | Zambia | 144 | Typhoid | May-13 | 2013 | unknown | unknown | contaminated water |
| 269 | Sub-Himalayan region, India | 31.684059, 76.301489 | Asia | Southern Asia | India | 43 | Paratyphoid | Mar-14 | 2014 | culture | no | unknown |
| 270 | Jorhat Assam, India | 26.760952, 94.211062 | Asia | Southeastern Asia | India | 79 | Typhoid | Jan-14 | 2014 | culture | no | waterborne |
| 271 | Tokyo, Japan | 35.705131, 139.732361 | Asia | Eastern Asia | Japan | 8 | Typhoid | Sep-14 | 2014 | culture | unknown | foodbourne |
| 272 | Saptari, Nepal | 26.628979, 86.705084 | Asia | Southern Asia | Nepal | 500 | Typhoid | Jul-14 | 2014 | unknown | unknown | contaminated water |
| 273 | Harare, Zimbabwe | -17.823501, 31.060978 | Africa |  | Zimbabwe | 18 | Typhoid | Jul-14 | 2014 | unknown | unknown | contaminated water |
| 274 | Bua, Fiji | -16.766282, 178.748344 | Oceania |  | Fiji | 24 | Typhoid | Jan-15 | 2015 | unknown | unknown | unknown |
| 275 | Sindhupalchok, Nepal | 27.920148, 85.728847 | Asia | Southern Asia | Nepal | 15 | Typhoid | Jul-15 | 2015 | culture | unknown | contaminated water |
| 276 | Damascus, Syria | 33.520909, 36.285640 | Asia | Western Asia | Syria | 90 | Typhoid | Aug-15 | 2015 | culture | unknown | unknown |
| 277 | Kampala- Wakiso- Mukono districts, Uganda | 0.331447, 32.591357 | Africa |  | Uganda | 12000 | Typhoid | Feb-15 | 2015 | culture | no | contaminated water |
| 278 | Colorado, USA | 38.996807, -105.690960 | North America |  | USA | 3 | Typhoid | Sep-15 | 2015 | culture | unknown | chronic carrier |
| 279 | Harare, Zimbabwe | -17.823501, 31.060978 | Africa |  | Zimbabwe | 600 | Typhoid | Mar-16 | 2016 | culture | unknown | contaminated water |
| 280 | Kigoma region | -4.887434, 29.659617 | Africa |  | Tanzania | 16 | Typhoid | May-15 | 2015 | culture | unknown | contaminated water |
| 281 | Kirehe District | -2.218112, 30.752247 | Africa |  | Rwanda | 1663 | Typhoid | Oct-15 | 2015 | culture | unknown | contaminated water |
| 282 | Bengaluru, India | 12.942215, 77.586939 | Asia | Southern Asia | India | 42 | Typhoid | Dec-15 | 2015 | culture | no | unknown |
| 283 | Hyderabad, Pakistan | 25.389767, 68.362900 | Asia | Southern Asia | Pakistan | 8188 | Typhoid | Nov-16 | 2016 | culture | yes | contaminated water |
| 284 | Auckland, New Zealnd | -36.883753, 174.752872 | Oceania |  | New Zealand | 20 | Typhoid | Mar-17 | 2017 | culture | unknown | person-person |
| 285 | al-Bab, Syria | 36.370353, 37.515600 | Asia | Western Asia | Syria | 20 | Typhoid | Mar-17 | 2017 | unknown | unknown | contaminated water |
| 286 | Veitongo, Tonga | -21.184991, -175.213479 | Oceania |  | Tonga | 11 | Typhoid | Apr-17 | 2017 | culture | unknown | unknown |
| 287 | Kalingalinga, Zambia | -15.402951, 28.330318 | Africa |  | Zambia | 55 | Typhoid | May-17 | 2017 | unknown | unknown | contaminated water |
| 288 | Yucatan, Mexico | 18.809228, -89.419696 | South America |  | Mexico | 110 | Typhoid | Jul-17 | 2017 | Confirm | no | unknown |
| 289 | Dolores, Guatemala | 16.511026, -89.415665 | Central America |  | Guatemala | 60 | Typhoid | Jul-17 | 2017 | unknown | unknown | contaminated water |
| 290 | Tramonti di Sopra, Italy | 46.309643, 12.789664 | Europe |  | Italy | 8 | Typhoid | Jul-17 | 2017 | culture | unknown | contaminated water |
| 291 | Krishna, India | 16.406932, 77.330833 | Asia | Southern Asia | India | 712 | Typhoid | Aug-17 | 2017 | unknown | unknown | contaminated water |
| 292 | Chungcheong, Korea | 36.619812, 127.368173 |  | Eastern Asia | Korea | 7 | Typhoid | Aug-17 | 2017 | culture | unknown | Imported |
| 293 | Wakapoa, Guyana | 7.516824, -58.799718 | South America |  | Guyana | 8 | Typhoid | Sep-17 | 2017 | unknown | unknown | unknown |
| 294 | Harare, Zimbabwe | -17.823501, 31.060978 | Africa |  | Zimbabwe | 3187 | Typhoid | Oct-17 | 2017 | culture | unknown | contaminated water |
| 295 | Ohio, USA | 40.202340, -83.025309 | North America |  | USA | 9 | Typhoid | Oct-17 | 2017 | confirmed unknown | unknown | contaminated water |
| 296 | Moturiki, Fiji | -17.764902, 178.742992 | Oceania |  | Fiji | 13 | Typhoid | Oct-17 | 2017 | unknown | unknown | unknown |
| 297 | Limpopo, South Africa | -23.906999, 29.419807 | Africa |  | South Africa | 60 | Typhoid | Nov-17 | 2017 | unknown | unknown | contaminated water |
| 298 | San Salvador, El Salvador | 13.693304, -89.213092 | Central America |  | El Salvador | 653 | Typhoid | Feb-18 | 2018 | unknown | unknown | contaminated water |
| 299 | Al Hol, Syria | 36.390895, 41.150494 | Asia | Western Asia | Syria | 269 | Typhoid | Mar-18 | 2018 | unknown | unknown | contaminated water |
| 300 | Massachusetts, USA | 42.355717, -72.167104 | North America |  | USA | 1 | Typhoid | May-18 | 2018 | unknown | unknown | imported |
| 301 | Gweru, Zimbabwe | -19.460146, 29.809340 | Africa |  | Zimbabwe | 355 | Typhoid | Aug-18 | 2018 | unknown | unknown | contaminated water |
| 302 | Trichy, India | 10.790872, 78.689218 | Asia | Southern Asia | India | 40 | Typhoid | Oct-18 | 2018 | unknown | unknown | contaminated water |
| 303 | Naitasiri, Fiji | -17.901574, 178.246556 | Oceania |  | Fiji | 31 | Typhoid | Dec-18 | 2018 | unknown | unknown | unknown |

| 1 | Styliads S, Borczyk A, 1994, Canada communicable disease report = Releve des maladies transmissibles au Canada, Typhoid outbreak associated with consumption of raw shellfish--Ontario., 20: 63-5 | |  |  |
| --- | --- | --- | --- | --- |
| 2 | el-Sherbini A, 1992, Journal of tropical pediatrics, An outbreak of typhoid fever resistant to chloramphenicol and other drugs in Gharbeya Governorate in Egypt., 38: 331-4 | |  |  |
| 3 | Ayyagari A, Pal N, 1991, Transactions of the Royal Society of Tropical Medicine and Hygiene, Outbreak of typhoid fever due to multiresistant Salmonella typhi in northern India--a preliminary report., 85: 302 | |  |  |
| 4 | Sarkar AK, Ganguly S, Ganguly S, 1991, Journal of the Indian Medical Association, Recent outbreak of chloramphenicol resistant typhoid fever in West Bengal., 89: 257-9 | |  |  |
| 5 | Ramanan A, Pandit N, Yeshwanth M, 1992, Indian pediatrics, Unusual complications in a multidrug resistant Salmonella typhi outbreak., 29: 118-20 | |  |  |
| 6 | Agarwal V, Jalgaonkar PD, Pathak AA, Saoji AM, 1992, The Journal of the Association of Physicians of India, An outbreak of multidrug resistant typhoid fever in Nagpur., 40: 416 | |  |  |
| 7 | Mishra S, Patwari AK, Anand VK, Pillai PK, Aneja S, Chandra J, Sharma D, 1992, Indian pediatrics, Multidrug resistant typhoid fever: therapeutic considerations., 29: 443-8 | |  |  |
| 8 | Sheorey HS, Kaundinya DV, Hulyalkar VS, Deshpande AK, 1993, Indian journal of pathology & microbiology, Multi drug resistant Salmonella typhi in Bombay., 36: 8-12 | |  |  |
| 9 | Figueroa JP, 1990, The West Indian medical journal, The typhoid fever outbreak in Jamaica., 39: 201-2 | |  |  |
| 10 | Figueroa JP, 1990, The West Indian medical journal, The typhoid fever outbreak in Jamaica., 39: 201-2 | |  |  |
| 11 | Thong KL, Cheong YM, Puthucheary S, Koh CL, Pang T, 1994, Journal of clinical microbiology, Epidemiologic analysis of sporadic Salmonella typhi isolates and those from outbreaks by pulsed-field gel electrophoresis., 32: 1135-41 | |  |  |
| 12 | Goh KT, Teo SH, Tay L, Monteiro EH, 1992, Epidemiology and infection, Epidemiology and control of an outbreak of typhoid in a psychiatric institution., 108: 221-9 | |  |  |
| 13 | Centers for Disease Control (CDC), 1990, MMWR. Morbidity and mortality weekly report, Typhoid fever--Skagit County, Washington., 39: 749-51 | |  |  |
| 14 | Mathieu JJ, Henning KJ, Bell E, Frieden TR, 1994, Archives of internal medicine, Typhoid fever in New York City, 1980 through 1990., 154: 1713-8 | |  |  |
| 15 | Cote TR, Convery H, Robinson D, Ries A, Barrett T, Frank L, Furlong W, Horan J, Dwyer D, 1995, Journal of community health, Typhoid fever in the park: epidemiology of an outbreak at a cultural interface., 20: 451-8 | |  |  |
| 16 | Olsen SJ, Bleasdale SC, Magnano AR, Landrigan C, Holland BH, Tauxe RV, Mintz ED, Luby S, 2003, Epidemiology and infection, Outbreaks of typhoid fever in the United States, 1960-99., 130: 13-21 | |  |  |
| 17 | Olsen SJ, Bleasdale SC, Magnano AR, Landrigan C, Holland BH, Tauxe RV, Mintz ED, Luby S, 2003, Epidemiology and infection, Outbreaks of typhoid fever in the United States, 1960-99., 130: 13-21 | |  |  |
| 18 | Verghese SL, Manonmani R, Balasubramanian S, Chandrasekharan S, 1992, The Journal of communicable diseases, Multi-drug resistance in salmonellae isolated from enteric fever cases at Porur--a semi urban area near Madras City., 24: 12-5 | |  |  |
| 19 | Mahanta J, 1994, Journal of the Indian Medical Association, Drug sensitivity of Salmonella paratyphi A isolated from a suspected outbreak of enteric fever in Duliajan., 92: 49-50 | |  |  |
| 20 | Biswal N, 1994, Journal of tropical pediatrics, Neurological manifestations of typhoid fever in children., 40: 190 | |  |  |
| 21 | Rathish KC, Chandrashekar MR, Nagesha CN, 1994, Indian journal of medical sciences, Multidrug resistant Salmonella typhi in Bangalore, south India., 48: 85-8 | |  |  |
| 22 | Rathish KC, Chandrashekar MR, Nagesha CN, 1995, Indian journal of pediatrics, An outbreak of multidrug resistant typhoid fever in Bangalore., 62: 445-8 | |  |  |
| 23 | Thong KL, Cheong YM, Puthucheary S, Koh CL, Pang T, 1994, Journal of clinical microbiology, Epidemiologic analysis of sporadic Salmonella typhi isolates and those from outbreaks by pulsed-field gel electrophoresis., 32: 1135-41 | |  |  |
| 24 | Coovadia YM, Gathiram V, Bhamjee A, Garratt RM, Mlisana K, Pillay N, Madlalose T, Short M, 1992, The Quarterly journal of medicine, An outbreak of multiresistant Salmonella typhi in South Africa., 82: 91-100 | |  |  |
| 25 | Usera MA, Aladuena A, Echeita A, Amor E, Gomez-Garces JL, Ibanez C, Mendez I, Sanz JC, Lopez-Brea M, 1993, European journal of epidemiology, Investigation of an outbreak of Salmonella typhi in a public school in Madrid., 9: 251-4 | |  |  |
| 26 | Reeve PA, Dwyer DE, 1995, The Medical journal of Australia, An outbreak of typhoid associated with a "lafet" in Vanuatu., 162: 55-6 | |  |  |
| 27 | Bradaric N, Punda-Polic V, Milas I, Ivic I, Grgic D, Radosevic N, Petric I, 1996, European journal of epidemiology, Two outbreaks of typhoid fever related to the war in Bosnia and Herzegovina., 12: 409-12 | |  |  |
| 28 | Bradaric N, Punda-Polic V, Milas I, Ivic I, Grgic D, Radosevic N, Petric I, 1996, European journal of epidemiology, Two outbreaks of typhoid fever related to the war in Bosnia and Herzegovina., 12: 409-12 | |  |  |
| 29 | Anonymous, 1993, World health forum, Typhoid fever alert in former Yugoslavia., 14: 204-5 | |  |  |
| 30 | Anonymous, 2000, , Typhoid fever epidemic in Samoa., 7: 35 | |  |  |
| 31 | al-Zubaidy AA, el Bushra HE, Mawlawi MY, 1995, East African medical journal, An outbreak of typhoid fever among children who attended a potluck dinner at Al-Mudhnab, Saudi Arabia., 72: 373-5 | |  |  |
| 32 | al-Quarawi SN, el Bushra HE, Fontaine RE, Bubshait SA, el Tantawy NA, 1995, Epidemiology and infection, Typhoid fever from water desalinized using reverse osmosis., 114: 41-50 | |  |  |
| 33 | Anonymous, 1992, Communicable disease report. CDR weekly, Typhoid fever in east London., 2: 141 | |  |  |
| 34 | Anonymous, 1994, Communicable disease report. CDR weekly, An outbreak of Salmonella paratyphi B in France., 4: 165 | |  |  |
| 35 | Sugiyama A, Nakano Y, Iwade Y, Yamauchi A, Sakurai N, Nakayama O, Yamamoto Y, Nakatsu M, Mori Y, Kishida Y, Oida T, Kumazawa NH, Terajima J, Nakamura A, 1999, Japanese journal of infectious diseases, Epidemiological studies of an outbreak of paratyphoid fever in the Shima area of Mie Prefecture., 52: 253-5 | |  |  |
| 36 | Rowe B, Ward LR, Threlfall EJ, 1997, Clinical infectious diseases : an official publication of the Infectious Diseases Society of America, Multidrug-resistant Salmonella typhi: a worldwide epidemic. [Review] [30 refs], 24 Suppl 1: S106-9 | |  |  |
| 37 | GIDEON online Outbreak-> 1993->Delmas, South Africa. 1993. | |  |  |
| 38 | McEvoy M, Susman MD, 1993, Communicable disease report. CDR review, Typhoid fever in north London., 3: R98-100 | |  |  |
| 39 | Olsen SJ, Bleasdale SC, Magnano AR, Landrigan C, Holland BH, Tauxe RV, Mintz ED, Luby S, 2003, Epidemiology and infection, Outbreaks of typhoid fever in the United States, 1960-99., 130: 13-21 | |  |  |
| 40 | Connerton P, Wain J, Hien TT, Ali T, Parry C, Chinh NT, Vinh H, Ho VA, Diep TS, Day NP, White NJ, Dougan G, Farrar JJ, 2000, Journal of clinical microbiology, Epidemic typhoid in vietnam: molecular typing of multiple-antibiotic-resistant Salmonella enterica serotype typhi from four outbreaks., 38: 895-7 | |  |  |
| 41 | Rahman M, Ahmad A, Shoma S, 2002, Epidemiology and infection, Decline in epidemic of multidrug resistant Salmonella typhi is not associated with increased incidence of antibiotic-susceptible strain in Bangladesh., 129: 29-34 | |  |  |
| 42 | Bradaric N., Smoljanovic M., Pavic S., Ivic I., Kalajdzic M., 1994, , Epidemiological and clinical characteristics of a typhoid fever outbreak in the Lasva Valley, Central Bosnia, spring 1994., 35: 177-182 | |  |  |
| 43 | Gruner E., Flepp M., Gabathuler U., Thong K.L., Altwegg M., 1997, , Outbreak of typhoid fever in a non-endemic area: Comparison of three molecular typing methods., 28: 179-185 | |  |  |
| 44 | Connerton P, Wain J, Hien TT, Ali T, Parry C, Chinh NT, Vinh H, Ho VA, Diep TS, Day NP, White NJ, Dougan G, Farrar JJ, 2000, Journal of clinical microbiology, Epidemic typhoid in vietnam: molecular typing of multiple-antibiotic-resistant Salmonella enterica serotype typhi from four outbreaks., 38: 895-7 | |  |  |
| 45 | Kulkarni AP, Powar RM, Mangalkar SM, Kulkarni VA, Nagalgaonkar RN, 1996, The Journal of communicable diseases, Epidemiological investigation of an outbreak of enteric fever in a village in Maharashtra., 28: 117-21 | |  |  |
| 46 | Fule RP, Ingole KV, Jalgaonkar SV, Moon BU, 1996, The Indian journal of medical research, Outbreak of food poisoning due to Salmonella paratyphi A var durazzo (2,12:a:-) in Yavatmal (Maharashtra) in May 1995., 103: 74-6 | |  |  |
| 47 | Bahrmand AR, Velayati AA, 1997, Scandinavian journal of infectious diseases, Antimicrobial resistance pattern and plasmid profile of Salmonella typhi isolated from an outbreak in Tehran province., 29: 265-9 | |  |  |
| 48 | Ghenghesh KS, Franka E, Tawil K, Wasfy MO, Ahmed SF, Rubino S, Klena JD, 2009, Journal of infection in developing countries, Enteric fever in Mediterranean north Africa. [Review] [74 refs], 3: 753-61 | |  |  |
| 49 | GIDEON online. Outbreak-> 1996->Northern Division, Fiji. 1996. | |  |  |
| 50 | Rampling A, 1996, BMJ (Clinical research ed.), Raw milk cheeses and Salmonella., 312: 67-8 | |  |  |
| 51 | Kapil A, Sood S, Reddaiah VP, Das B, Seth P, 1997, Emerging infectious diseases, Paratyphoid fever due to Salmonella enterica serotype Paratyphi A., 3: 407 | |  |  |
| 52 | Yoo S et al, 2004, Journal of Korean medical science, Epidemiology of Salmonella enterica serotype typhi infections in Korea for recent 9 years: trends of antimicrobial resistance., 19: 15-20 | |  |  |
| 53 | Yoo S et al, 2004, Journal of Korean medical science, Epidemiology of Salmonella enterica serotype typhi infections in Korea for recent 9 years: trends of antimicrobial resistance., 19: 15-20 | |  |  |
| 54 | Ty AU, Ang GY, Ang LW, James L, Goh KT, 2010, Annals of the Academy of Medicine, Singapore, Changing epidemiology of enteric fevers in Singapore., 39: 889-8 | |  |  |
| 55 | ProMED-mail. Typhoid Fever in Taiwan http://www.promedmail.org. [cited ProMED-mail archive:19961010.1718] | |  |  |
| 56 | Mermin J.H. et al., 1999, , A massive epidemic of multidrug-resistant typhoid fever in Tajikistan associated with consumption of municipal water., 179: 1416-1422 | |  |  |
| 57 | ProMED-mail. Typhoid Fever in Kulyab, Tajikistan http://www.promedmail.org. [cited ProMED-mail archive:19970221.0416] | |  |  |
| 58 | Pancharoen C., Thisyakom U., 1998, , Paratyphoid fever in Thai children: An eleven-year experience., 7: 106-108 | |  |  |
| 59 | Olsen SJ, Bleasdale SC, Magnano AR et al, 2003, Epidemiology and infection, Outbreaks of typhoid fever in the United States, 1960-99., 130: 13-21 | |  |  |
| 60 | ProMED-mail. Typhoid Fever in Juan Dolio, Dominican Republic http://www.promedmail.org. [cited ProMED-mail archive:19970815.1723] | |  |  |
| 61 | Pradier C, Keita-Perse O, Bernard E, Gisbert C, 2000, European journal of clinical microbiology & infectious diseases : official publication of the European Society of Clinical Microbiology, Outbreak of typhoid fever on the French Riviera., 19: 464-7 | |  |  |
| 62 | ProMED-mail. Typhoid Fever in Ashkabad, Turkmenistan http://www.promedmail.org. [cited ProMED-mail archive:19970311.0545] | |  |  |
| 63 | ProMED-mail. Typhoid Fever in Glamorgan, Wales http://www.promedmail.org. [cited ProMED-mail archive:19970314.0563] | |  |  |
| 64 | GIDEON online. Outbreak-> 1998->Armenia. 1998. | |  |  |
| 65 | Valenciano M, Baron S, Fisch A, Grimont F, Desenclos JC, 2000, American journal of epidemiology, Investigation of concurrent outbreaks of gastroenteritis and typhoid fever following a party on a floating restaurant, France, March 1998., 152: 934-9 | |  |  |
| 66 | Olsen SJ, Kafoa B, Win NS et al, 2001, Epidemiology and infection, Restaurant-associated outbreak of Salmonella typhi in Nauru: an epidemiological and cost analysis., 127: 405-12 | |  |  |
| 67 | Katz DJ, Cruz MA, Trepka MJ et al, 2002, The Journal of infectious diseases, An outbreak of typhoid Fever in Florida associated with an imported frozen fruit., 186: 234-9 | |  |  |
| 68 | ProMED-mail. Typhoid Fever in Vanadzor, Armenia http://www.promedmail.org. [cited ProMED-mail archive:19990504.073] | |  |  |
| 69 | ProMED-mail. Typhoid Fever in Austrailia http://www.promedmail.org. [cited ProMED-mail archive:19990608.0977] | |  |  |
| 70 | Hang Hui Yang, Kilgore P.E., Ling Hong Yang et al., 2001, , An outbreak of typhoid fever, Xing-An County, People's Republic of China, 1999: Estimation of the field effectiveness of Vi polysaccharide typhoid vaccine., 183: 1775-1780 | |  |  |
| 71 | ProMED-mail. Typhoid Fever in Zugdidi, Georgia http://www.promedmail.org. [cited ProMED-mail archive:19990428.0702] | |  |  |
| 72 | ProMED-mail. Typhoid Fever in Guatemala city, Guatemala http://www.promedmail.org. [cited ProMED-mail archive:19990420.0648] | |  |  |
| 73 | ProMED-mail. Typhoid Fever in Izabal, Guatemala http://www.promedmail.org. [cited ProMED-mail archive:19990420.0648] | |  |  |
| 74 | Rajeev A, 1999, The Journal of communicable diseases, Quinolone resistant typhoid outbreak in an extended joint family., 31: 263-5 | |  |  |
| 75 | John TJ, Rajappan K, Arjunan KK, 2004, The Indian journal of medical research, Communicable diseases monitored by disease surveillance in Kottayam district, Kerala state, India., 120: 86-93 | |  |  |
| 76 | ProMED-mail. Typhoid Fever in Tokyo, Japan http://www.promedmail.org. [cited ProMED-mail archive:19990411.06] | |  |  |
| 77 | ProMED-mail. Typhoid Fever in Kavieng town, Papa New Guinea http://www.promedmail.org. [cited ProMED-mail archive:19990112.0042] | |  |  |
| 78 | Ferson M.J., Ressler K.-A., 2005, , Bound for Sydney town: Health surveillance on international cruise vessels visiting the Port of Sydney., 182: 391-394 | |  |  |
| 79 | ProMED-mail. Typhoid Fever in Krasnoyarsk, Russia http://www.promedmail.org. [cited ProMED-mail archive:19990320.0443] | |  |  |
| 80 | Swaddiwudhipong W, Kanlayanaphotporn J, 2001, Journal of the Medical Association of Thailand = Chotmaihet thangphaet, A common-source water-borne outbreak of multidrug-resistant typhoid fever in a rural Thai community., 84: 1513-7 | |  |  |
| 81 | ProMED-mail. Typhoid Fever in Nuku'alofa, Tonga http://www.promedmail.org. [cited ProMED-mail archive:19990408.0576] | |  |  |
| 82 | Grewal HM, Jureen R, Steinsland H, Digranes A, 2002, Scandinavian journal of infectious diseases, Molecular epidemiological study of Salmonella enterica serovar paratyphi B infections imported from Turkey to Western Norway., 34: 5-10 | |  |  |
| 83 | Salve A, Pichel M, Wiesner M et al, 2006, Foodborne pathogens and disease, Molecular subtyping of Salmonella enterica serovar Typhi isolates from Colombia and Argentina., 3: 142-52 | |  |  |
| 84 | Misra R.N.,Bawa K.S., Magu S.K. et al, 2005, , Outbreak of multi-drug resistant Salmonella typhi enteric fever in Mumbai garrison., 61: 148-150 | |  |  |
| 85 | ProMED-mail. Typhoid Fever in Almaty, Khazakstan http://www.promedmail.org. [cited ProMED-mail archive:20001116.2006] | |  |  |
| 86 | ProMED-mail. Typhoid Fever in Hammana, Lebanon http://www.promedmail.org. [cited ProMED-mail archive:20000327.0449] | |  |  |
| 87 | Aye TT, Siriarayapon P, 2004, Journal of the Medical Association of Thailand = Chotmaihet thangphaet, Typhoid fever outbreak in Madaya Township, Mandalay Division, Myanmar, September 2000., 87: 395-9 | |  |  |
| 88 | ProMED-mail. Typhoid Fever in Shakhtyorsk, Russia http://www.promedmail.org. [cited ProMED-mail archive:20000504.0683] | |  |  |
| 89 | ProMED-mail. Typhoid Fever in Yasnogorsk, Russia http://www.promedmail.org. [cited ProMED-mail archive:20001026.1859] | |  |  |
| 90 | Anonymous, 2000, , Typhoid fever epidemic in Samoa., 7: 35 | |  |  |
| 91 | ProMED-mail. Typhoid Fever in Jizan, Saudi Arabia http://www.promedmail.org. [cited ProMED-mail archive:20001215.2196] | |  |  |
| 92 | Reller ME, Olsen SJ, Kressel AB et al, 2003, Clinical infectious diseases : an official publication of the Infectious Diseases Society of America, Sexual transmission of typhoid fever: a multistate outbreak among men who have sex with men., 37: 141-4 | |  |  |
| 93 | Yoon J, Segal-Maurer S, Rahal JJ, 2004, Archives of internal medicine, An outbreak of domestically acquired typhoid fever in Queens, NY., 164: 565-7 | |  |  |
| 94 | Michel R, Garnotel E, Spiegel A et al, 2005, European journal of epidemiology, Outbreak of typhoid fever in vaccinated members of the French Armed Forces in the Ivory Coast., 20: 635-42 | |  |  |
| 95 | Kariuki S, Revathi G, Muyodi J et al, 2004, Journal of clinical microbiology, Characterization of multidrug-resistant typhoid outbreaks in Kenya., 42: 1477-82 | |  |  |
| 96 | Kariuki S, Revathi G, Muyodi J et al, 2004, Journal of clinical microbiology, Characterization of multidrug-resistant typhoid outbreaks in Kenya., 42: 1477-82 | |  |  |
| 97 | Kariuki S, Revathi G, Muyodi J et al, 2004, Journal of clinical microbiology, Characterization of multidrug-resistant typhoid outbreaks in Kenya., 42: 1477-82 | |  |  |
| 98 | ProMED-mail. Typhoid Fever in Nakuru, Kenya http://www.promedmail.org. [cited ProMED-mail archive:20010606.1113] | |  |  |
| 99 | ProMED-mail. Typhoid Fever in Batken, Kyrgyzstan http://www.promedmail.org. [cited ProMED-mail archive:20010816.1935] | |  |  |
| 100 | Ansari I, Adhikari N, Pandey R et al, 2005, Tropical doctor, Enteric fever: is ciprofloxacin failing in Nepal?., 35: 57-8 | |  |  |
| 101 | GIDEON online. Outbreak-> 2001->Gwent, UK. 2001. | |  |  |
| 102 | GIDEON online. Outbreak-> 2001->Newport, Wales. 2001. | |  |  |
| 103 | Hinman SE, Blackburn JK, Curtis A, 2006, International journal of health geographics, Spatial and temporal structure of typhoid outbreaks in Washington, D.C., 1906-1909: evaluating local clustering with the Gi* statistic., 5: 13 | |  |  |
| 104 | ProMED-mail. Typhoid Fever in Mombasa, Kenya http://www.promedmail.org. [cited ProMED-mail archive:20020524.4312] | |  |  |
| 105 | Kim S, 2010, Journal of Korean medical science, Salmonella serovars from foodborne and waterborne diseases in Korea, 1998-2007: total isolates decreasing versus rare serovars emerging. [Review], 25: 1693-9 | |  |  |
| 106 | Lewis MD, Serichantalergs O, Pitarangsi C et al, 2005, Clinical infectious diseases : an official publication of the Infectious Diseases Society of America, Typhoid fever: a massive, single-point source, multidrug-resistant outbreak in Nepal., 40: 554-61 | |  |  |
| 107 | ProMED-mail. Typhoid Fever in Bambang, Philippeans http://www.promedmail.org. [cited ProMED-mail archive:20020722.4839] | |  |  |
| 108 | ProMED-mail. Typhoid Fever in Samoa http://www.promedmail.org. [cited ProMED-mail archive:20021031.5673] | |  |  |
| 109 | Wang JL, Kao JH, Tseng SP et al, 2005, Epidemiology and infection, Typhoid fever and typhoid hepatitis in Taiwan., 133: 1073-9 | |  |  |
| 110 | ProMED-mail. Typhoid Fever in Murghob, Tajikistan http://www.promedmail.org. [cited ProMED-mail archive: 20021209.6026] | |  |  |
| 111 | Weill FX, Tran HH, Roumagnac P et al, 2007, The American journal of tropical medicine and hygiene, Clonal reconquest of antibiotic-susceptible Salmonella enterica serotype Typhi in Son La Province, Vietnam., 76: 1174-81 | |  |  |
| 112 | Weill FX, Tran HH,Roumagnac P et al, 2007, The American journal of tropical medicine and hygiene, Clonal reconquest of antibiotic-susceptible Salmonella enterica serotype Typhi in Son La Province, Vietnam., 76: 1174-81 | |  |  |
| 113 | Weill FX, Tran HH, Roumagnac P et al, 2007, The American journal of tropical medicine and hygiene, Clonal reconquest of antibiotic-susceptible Salmonella enterica serotype Typhi in Son La Province, Vietnam., 76: 1174-81 | |  |  |
| 114 | ProMED-mail. Typhoid Fever in Megri, Armenia http://www.promedmail.org. [cited ProMED-mail archive:20030922.2389] | |  |  |
| 115 | Yu F, Fan S, Fan X et al, 2011, European journal of clinical microbiology & infectious diseases : official publication of the European Society of Clinical Microbiology, Analysis of characteristics of paratyphoid A in 157 Chinese inpatients between 1998 and 2009., 30: 71-5 | |  |  |
| 116 | ProMED-mail. Typhoid Fever in Paris, France http://www.promedmail.org. [cited ProMED-mail archive:20031117.2852] | |  |  |
| 117 | ProMED-mail. Typhoid Fever in Ashanti, Ghana http://www.promedmail.org. [cited ProMED-mail archive:20031208.3013] | |  |  |
| 118 | GIDEON online. Outbreak-> 2003->Grand Bois, Haiti. 2003. | |  |  |
| 119 | Banerjee A., Kalghatgi A.T., Singh P.M.P. et al., 2007, , Epidemiological investigation of an outbreak of enteric fever., 63: 322-324 | |  |  |
| 120 | Ranjbar R, Salimkhani E, Sadeghifard N et al, 2007, Pakistan journal of biological sciences : PJBS, An outbreak of gastroenteritis of unknown origin in Tehran, July 2003., 10: 1138-40 | |  |  |
| 121 | ProMED-mail. Typhoid Fever in Batken, Kyrgyzstan http://www.promedmail.org. [cited ProMED-mail archive:20031031.271] | |  |  |
| 122 | ProMED-mail. Typhoid Fever in Bacoor, Philippines http://www.promedmail.org. [cited ProMED-mail archive:20031223.3122] | |  |  |
| 123 | ProMED-mail. Typhoid Fever in Dushanbe, Tajikistan http://www.promedmail.org. [cited ProMED-mail archive:20031024.2662] | |  |  |
| 124 | ProMED-mail. Typhoid Fever in Batna, Algeria http://www.promedmail.org. [cited ProMED-mail archive:20040902.2444] | |  |  |
| 125 | Hamner S, Tripathi A, Mishra RK et al, 2006, International journal of environmental health research, The role of water use patterns and sewage pollution in incidence of water-borne/enteric diseases along the Ganges river in Varanasi, India., 16: 113-32 | |  |  |
| 126 | Kato Y, Fukayama M, Adachi T et al, 2007, Emerging infectious diseases, Multidrug-resistant typhoid fever outbreak in travelers returning from Bangladesh., 13: 1954-5 | |  |  |
| 127 | Yang HH, Gong J, Zhang J et al, 2010, Epidemiology and infection, An outbreak of Salmonella Paratyphi A in a boarding school: a community-acquired enteric fever and carriage investigation., 138: 1765-74 | |  |  |
| 128 | ProMED-mail. Typhoid Fever in Famagusta, Cyprus http://www.promedmail.org. [cited ProMED-mail archive:20040813.2242] | |  |  |
| 129 | Anonymous, 2005, , Typhoid fever in Democratic Republic of the Congo., 25: 76 | |  |  |
| 130 | ProMED-mail. Typhoid Fever in Westmoreland, Jamaica http://www.promedmail.org. [cited ProMED-mail archive:20040219.0532] | |  |  |
| 131 | Al-Sanouri TM, Paglietti B, Haddadin A et al, 2008, Journal of infection in developing countries, Emergence of plasmid-mediated multidrug resistance in epidemic and non-epidemic strains of Salmonella enterica serotype Typhi from Jordan., 2: 295-301 | |  |  |
| 132 | ProMED-mail. Typhoid Fever in Batken, Kyrgyzstan http://www.promedmail.org. [cited ProMED-mail archive:20040712.1871] | |  |  |
| 133 | ProMED-mail. Typhoid Fever in Jalal-Abad, Kyrgyzstan http://www.promedmail.org. [cited ProMED-mail archive:20041022.2856] | |  |  |
| 134 | ProMED-mail. Typhoid Fever in Osh City, Kyrgzstan http://www.promedmail.org. [cited ProMED-mail archive:20041101.2952] | |  |  |
| 135 | ProMED-mail. Typhoid Fever in Talas, Kyrgyzstan http://www.promedmail.org. [cited ProMED-mail archive:20040809.2198] | |  |  |
| 136 | Farooqui A, Khan A, Kazmi SU, 2009, BMC public health, Investigation of a community outbreak of typhoid fever associated with drinking water., 9: 476 | |  |  |
| 137 | ProMED-mail. Typhoid Fever in Morobe, Papa New Guinea http://www.promedmail.org. [cited ProMED-mail archive:20040308.0652] | |  |  |
| 138 | ProMED-mail. Typhoid Fever in Komi, Russia http://www.promedmail.org. [cited ProMED-mail archive:20040316.0726] | |  |  |
| 139 | ProMED-mail. Typhoid Fever in Moscow, Russia http://www.promedmail.org. [cited ProMED-mail archive:20040728.2059] | |  |  |
| 140 | ProMED-mail. Typhoid Fever in Ruhengeri, Rwanda http://www.promedmail.org. [cited ProMED-mail archive:20040702.1768] | |  |  |
| 141 | ProMED-mail. Typhoid Fever in Jaffna, Sri Lanka http://www.promedmail.org. [cited ProMED-mail archive:20040727.205] | |  |  |
| 142 | ProMED-mail. Typhoid Fever in Dushanbe, Tajikistan http://www.promedmail.org. [cited ProMED-mail archive:20040722.1998] | |  |  |
| 143 | ProMED-mail. Typhoid Fever in Hatlonskaya, Tajikistan http://www.promedmail.org. [cited ProMED-mail archive:20040628.1725] | |  |  |
| 144 | ProMED-mail. Typhoid Fever in Kolkhozabad, Tajikistan http://www.promedmail.org. [cited ProMED-mail archive: 20041219.3351] | |  |  |
| 145 | ProMED-mail. Typhoid Fever in Shahrinav, Tajikistan http://www.promedmail.org. [cited ProMED-mail archive:20040621.1654] | |  |  |
| 146 | Ben Saida N, Mhalla S, Bouzouia N, Boukadida J, 2007, Pathologie-biologie, Genotypic analysis of Salmonella enterica serovar Typhi collected during two successive autumnal typhoid outbreaks in southeast Tunisia., 55: 336-9 | |  |  |
| 147 | ProMED-mail. Typhoid Fever in Odessa, Ukraine http://www.promedmail.org. [cited ProMED-mail archive:20040721.1986] | |  |  |
| 148 | Holt KE, Dolecek C, Chau TT et al, 2011, PLoS neglected tropical diseases, Temporal fluctuation of multidrug resistant salmonella typhi haplotypes in the mekong river delta region of Vietnam., 5: e929 | |  |  |
| 149 | Khezzani B., Bouchemal S., 2016, , A study of epidemic of typhoid fever in the Souf oasis (eastern south of Algeria)., 7: 1299-1307 | |  |  |
| 150 | ProMED-mail. Typhoid Fever in Central/ Eastern Divisions, Fiji http://www.promedmail.org. [cited ProMED-mail archive:20050818.242] | |  |  |
| 151 | ProMED-mail. Typhoid Fever in Wainunu, Fiji http://www.promedmail.org. [cited ProMED-mail archive:20050818.242] | |  |  |
| 152 | GIDEON online Outbreak-> 2005->French Guiana. 2005., , , , : | |  |  |
| 153 | ProMED-mail. Typhoid Fever in Oyem, Gabon http://www.promedmail.org. [cited ProMED-mail archive:20050107.0052] | |  |  |
| 154 | ProMED-mail. Typhoid Fever in Yuen Long District, Hong Kong http://www.promedmail.org. [cited ProMED-mail archive:20060110.0089] | |  |  |
| 155 | Dhadwal B.S., Shetty R.A., 2008, , Epidemiological investigation of a typhoid outbreak., 64: 241-242 | |  |  |
| 156 | ProMED-mail. Typhoid Fever in Bungoma, Kenya http://www.promedmail.org. [cited ProMED-mail archive:20050321.0816] | |  |  |
| 157 | ProMED-mail. Typhoid Fever in Kara-Suu, Kyrgyzstan http://www.promedmail.org. [cited ProMED-mail archive:20050627.1813] | |  |  |
| 158 | ProMED-mail. Typhoid Fever in Kyrgyzstan http://www.promedmail.org. [cited ProMED-mail archive:20050912.27] | |  |  |
| 159 | Baddam R, Kumar N, Shaik S, Lankapalli AK, Ahmed N, 2014, Scientific reports, Genome dynamics and evolution of Salmonella Typhi strains from the typhoid-endemic zones., 4: 7457 | |  |  |
| 160 | ProMED-mail. Typhoid Fever in Borbon, Philippines http://www.promedmail.org. [cited ProMED-mail archive:20051027.313] | |  |  |
| 161 | ProMED-mail. Typhoid Fever in Dagupenos, Philippines http://www.promedmail.org. [cited ProMED-mail archive:20050921.2789] | |  |  |
| 162 | ProMED-mail. Typhoid Fever in Pasay, Philippines http://www.promedmail.org. [cited ProMED-mail archive:20050202.0354] | |  |  |
| 163 | ProMED-mail. Typhoid Fever in St. Petersburg, Russia http://www.promedmail.org. [cited ProMED-mail archive:20060110.0089] | |  |  |
| 164 | Keddy KH, Sooka A, Ismail H et al, 2011, Epidemiology and infection, Molecular epidemiological investigation of a typhoid fever outbreak in South Africa, 2005: the relationship to a previous epidemic in 1993., 139: 1239-45 | |  |  |
| 165 | ProMED-mail. Typhoid Fever in Nongoma, South Africa http://www.promedmail.org. [cited ProMED-mail archive:20051027.313] | |  |  |
| 166 | ProMED-mail. Typhoid Fever in Transkei, South Africa http://www.promedmail.org. [cited ProMED-mail archive:20050509.1275] | |  |  |
| 167 | Chiou CS  Wei HL, Mu JJ, Liao YS et al, 2013, Journal of clinical microbiology, Salmonella enterica serovar Typhi variants in long-term carriers., 51: 669-72 | |  |  |
| 168 | ProMED-mail. Typhoid Fever in Zakarpatye, Ukraine http://www.promedmail.org. [cited ProMED-mail archive:20051219.3637] | |  |  |
| 169 | GIDEON online. Outbreak-> 2005->New York, USA. 2005. | |  |  |
| 170 | ProMED-mail. Typhoid Fever in Yunnan, China http://www.promedmail.org. [cited ProMED-mail archive:20060828.2449] | |  |  |
| 171 | ProMED-mail. Typhoid Fever in Northern Divison, Fiji http://www.promedmail.org. [cited ProMED-mail archive:20060828.2449] | |  |  |
| 172 | ProMED-mail. Typhoid Fever in Kerala, India http://www.promedmail.org. [cited ProMED-mail archive:20060828.2449] | |  |  |
| 173 | ProMED-mail. Typhoid Fever in Jalal-Abad, Kyrgyzstan http://www.promedmail.org. [cited ProMED-mail archive:20060222.0572] | |  |  |
| 174 | ProMED-mail. Typhoid Fever in Lebanon http://www.promedmail.org. [cited ProMED-mail archive:20061121.3315] | |  |  |
| 175 | ProMED-mail. Typhoid Fever in Majuro, Marshall Islands http://www.promedmail.org. [cited ProMED-mail archive:20060629.1799] | |  |  |
| 176 | ProMED-mail. Typhoid Fever in Manthali, Nepal http://www.promedmail.org. [cited ProMED-mail archive:20061106.3188] | |  |  |
| 177 | ProMED-mail. Typhoid Fever in Sankhuwasabha, Nepal http://www.promedmail.org. [cited ProMED-mail archive:20061121.3315] | |  |  |
| 178 | ProMED-mail. Typhoid Fever in Erave, Papa New Guinea http://www.promedmail.org. [cited ProMED-mail archive:20060411.1077] | |  |  |
| 179 | ProMED-mail. Typhoid Fever in Isabela, Philippines http://www.promedmail.org. [cited ProMED-mail archive:20060411.1077] | |  |  |
| 180 | ProMED-mail. Typhoid Fever in Mindanao, Philippines http://www.promedmail.org. [cited ProMED-mail archive:20061106.3188] | |  |  |
| 181 | ProMED-mail. Typhoid Fever in St. Petersburg, Russia http://www.promedmail.org. [cited ProMED-mail archive:20061106.3188] | |  |  |
| 182 | ProMED-mail. Typhoid Fever in Limpopo, South Africa http://www.promedmail.org. [cited ProMED-mail archive:20060222.0572] | |  |  |
| 183 | ProMED-mail. Typhoid Fever in Charsada, Afghanistan http://www.promedmail.org. [cited ProMED-mail archive:20070215.057] | |  |  |
| 184 | Khezzani B., Bouchemal S., 2016, , A study of epidemic of typhoid fever in the Souf oasis (eastern south of Algeria)., 7: 1299-1307 | |  |  |
| 185 | ProMED-mail. Typhoid Fever in Djelfa, Algeria http://www.promedmail.org. [cited ProMED-mail archive:20070829.2842] | |  |  |
| 186 | ProMED-mail. Typhoid Fever in Jujuy, Argentina http://www.promedmail.org. [cited ProMED-mail archive:20070116.0205] | |  |  |
| 187 | ProMED-mail. Typhoid Fever in Armenia http://www.promedmail.org. [cited ProMED-mail archive:20070921.3143] | |  |  |
| 188 | ProMED-mail. Typhoid Fever in Northern Divison, Fiji http://www.promedmail.org. [cited ProMED-mail archive:20070412.1226] | |  |  |
| 189 | ProMED-mail. Typhoid Fever in Northern Divison, Fiji http://www.promedmail.org. [cited ProMED-mail archive:20071203.3893], , , , : | |  |  |
| 190 | Bhunia R, Hutin Y, Ramakrishnan R, Pal N, Sen T, Murhekar M, 2009, BMC public health, A typhoid fever outbreak in a slum of South Dumdum municipality, West Bengal, India, 2007: evidence for foodborne and waterborne transmission., 9: 115 | |  |  |
| 191 | Anand PK, Ramakrishnan R, 2010, The Indian journal of medical research, Investigation of the outbreak of typhoid in a village of Thar Desert Rajasthan, India., 131: 799-803 | |  |  |
| 192 | ProMED-mail. Typhoid Fever in Hyderabad, India http://www.promedmail.org. [cited ProMED-mail archive:20070716.2286] | |  |  |
| 193 | ProMED-mail. Typhoid Fever in Manipur, India http://www.promedmail.org. [cited ProMED-mail archive:20070627.2072] | |  |  |
| 194 | ProMED-mail. Typhoid Fever in Majuro, Marshall Islands http://www.promedmail.org. [cited ProMED-mail archive:20070315.0912] | |  |  |
| 195 | ProMED-mail. Typhoid Fever in Baglung, Nepal http://www.promedmail.org. [cited ProMED-mail archive:20070627.2072] | |  |  |
| 196 | Ruscoe Q., Thompson N., van der Pol P et al, 2007, , Outbreak case reports., 5: 5-7 | |  |  |
| 197 | ProMED-mail. Typhoid Fever in Manukau, New Zealand http://www.promedmail.org. [cited ProMED-mail archive:20070119.0254] | |  |  |
| 198 | ProMED-mail. Typhoid Fever in Islamabad, Pakistan http://www.promedmail.org. [cited ProMED-mail archive:20070627.2072] | |  |  |
| 199 | ProMED-mail. Typhoid Fever in Daru, Papa New Guinea http://www.promedmail.org. [cited ProMED-mail archive:20071203.3893] | |  |  |
| 200 | ProMED-mail. Typhoid Fever in Enga, Papa New Guinea http://www.promedmail.org. [cited ProMED-mail archive:20070905.2928] | |  |  |
| 201 | ProMED-mail. Typhoid Fever in Caraga, Philippines http://www.promedmail.org. [cited ProMED-mail archive:20070123.0301] | |  |  |
| 202 | ProMED-mail. Typhoid Fever in Catanduanes, Philippines http://www.promedmail.org. [cited ProMED-mail archive:20080128.0358] | |  |  |
| 203 | ProMED-mail. Typhoid Fever in Mindanao, Philippines http://www.promedmail.org. [cited ProMED-mail archive:20070412.1226] | |  |  |
| 204 | ProMED-mail. Typhoid Fever in Moscow, Russia http://www.promedmail.org. [cited ProMED-mail archive:20070123.0301] | |  |  |
| 205 | ProMED-mail. Typhoid Fever in Mus, Turkey http://www.promedmail.org. [cited ProMED-mail archive:20070315.0912] | |  |  |
| 206 | Neil KP, Sodha SV, Lukwago L et al, 2012, Clinical infectious diseases : an official publication of the Infectious Diseases Society of America, A large outbreak of typhoid fever associated with a high rate of intestinal perforation in Kasese District, Uganda, 2008-2009., 54: 1091-9 | |  |  |
| 207 | ProMED-mail. Typhoid Fever in Hubei, China http://www.promedmail.org. [cited ProMED-mail archive:20081229.4095] | |  |  |
| 208 | ProMED-mail. Typhoid Fever in Northern Divison, Fiji http://www.promedmail.org. [cited ProMED-mail archive:20080204.045] | |  |  |
| 209 | Shaikhani MA, Husein HA, Karbuli TA, Mohamed MA, 2013, Indian journal of gastroenterology : official journal of the Indian Society of Gastroenterology, Colonoscopic findings and management of patients with outbreak typhoid fever presenting with lower gastrointestinal bleeding., 32: 335-40 | |  |  |
| 210 | Azmani W., Rosemawati A., Rohan D., Wan Mansor H., 2010, , Typhoid in Chuchuh Puteri Village, Kelantan., 65: 92 | |  |  |
| 211 | Ruscoe Q., 2009, , Outbreak case reports., 7: 6-7 | |  |  |
| 212 | ProMED-mail. Typhoid Fever in Milne Bay, Papa New Guinea http://www.promedmail.org. [cited ProMED-mail archive:20081229.4095] | |  |  |
| 213 | ProMED-mail. Typhoid Fever in Iloilo, Philippines http://www.promedmail.org. [cited ProMED-mail archive:20080312.0999] | |  |  |
| 214 | ProMED-mail. Typhoid Fever in Kisulad, Philippines http://www.promedmail.org. [cited ProMED-mail archive:20080128.0358] | |  |  |
| 215 | ProMED-mail. Typhoid Fever in Laguna, Philippines http://www.promedmail.org. [cited ProMED-mail archive:20080303.0875] | |  |  |
| 216 | ProMED-mail. Typhoid Fever in Nueva Vizcaya, Philippines http://www.promedmail.org. [cited ProMED-mail archive:20080128.0358] | |  |  |
| 217 | ProMED-mail. Typhoid Fever in Quezon, Philippines http://www.promedmail.org. [cited ProMED-mail archive:20081118.364] | |  |  |
| 218 | Aypak A, Celik AK, Aypak C, Cikman O, 2010, Tropical doctor, Multidrug resistant typhoid fever outbreak in Ercek Village-Van, Eastern Anatolia, Turkey: clinical profile, sensitivity patterns and response to antimicrobials., 40: 160-2 | |  |  |
| 219 | ProMED-mail. Typhoid Fever in Taveuni, Fiji http://www.promedmail.org. [cited ProMED-mail archive:20090710.2472] | |  |  |
| 220 | Singla N, Bansal N, Gupta V, Chander J, 2013, Asian Pacific journal of tropical medicine, Outbreak of Salmonella Typhi enteric fever in sub-urban area of North India: a public health perspective., 6: 167-8 | |  |  |
| 221 | Anita S, Amir KM, Fadzilah K et al, 2012, The Medical journal of Malaysia, Risk factors for typhoid outbreak in Sungai Congkak Recreational Park, Selangor 2009., 67: 12-6 | |  |  |
| 222 | Blum L.S., Dentz H., Chingoli F. et al., 2011, , Formative assessment of acceptability of typhoid vaccine in Neno District, Malawi., 85: 26 | |  |  |
| 223 | Gal-Mor O, Suez J, Elhadad D et al, 2012, Clinical and vaccine immunology : CVI, Molecular and cellular characterization of a Salmonella enterica serovar Paratyphi a outbreak strain and the human immune response to infection., 19: 146-56 | |  |  |
| 224 | Limpitikul W, Henpraserttae N, Saksawad R, Laoprasopwattana K, 2014, PloS one, Typhoid outbreak in Songkhla, Thailand 2009-2011: clinical outcomes, susceptibility patterns, and reliability of serology tests., 9: e111768 | |  |  |
| 225 | Walters MS, Routh J, Mikoleit M et al, 2014, PLoS neglected tropical diseases, Shifts in geographic distribution and antimicrobial resistance during a prolonged typhoid fever outbreak--Bundibugyo and Kasese Districts, Uganda, 2009-2011., 8: e2726 | |  |  |
| 226 | Okui S.A., 2014, , Persistent typhoid fever epidemics in internally displaced persons camps in Uganda., 91: 128-129 | |  |  |
| 227 | ProMED-mail. Typhoid Fever in Tennessee, USA http://www.promedmail.org. [cited ProMED-mail archive:20090608.212] | |  |  |
| 228 | Yan M  Yang B, Wang Z, Wang S et al, 2015, PLoS neglected tropical diseases, A Large-Scale Community-Based Outbreak of Paratyphoid Fever Caused by Hospital-Derived Transmission in Southern China., 9: e0003859 | |  |  |
| 229 | Yan M, Li X, Liao Q et al, B, 2016, Emerging microbes & infections, The emergence and outbreak of multidrug-resistant typhoid fever in China., 5: e62 | |  |  |
| 230 | ProMED-mail. Typhoid Fever in GUANGXI ZHUANG, China http://www.promedmail.org. [cited ProMED-mail archive:20101101.396] | |  |  |
| 231 | ProMED-mail. Typhoid Fever in Northern Divison, Fiji http://www.promedmail.org. [cited ProMED-mail archive:20100309.0762] | |  |  |
| 232 | GIDEON online. Outbreak-> 2010->Andhra Pradesh, India. 2010. | |  |  |
| 233 | ProMED-mail. Typhoid Fever in Haryana, India http://www.promedmail.org. [cited ProMED-mail archive:20100829.3076] | |  |  |
| 234 | ProMED-mail. Typhoid Fever in Russia (Far East) http://www.promedmail.org. [cited ProMED-mail archive:20100111.0125] | |  |  |
| 235 | Smith AM, Keddy KH, Ismail H et al, Group for Enteric, Respiratory and Meningeal Disease Surveillance in South Africa (GERMS-SA), 2011, Journal of medical microbiology, International collaboration tracks typhoid fever cases over two continents from South Africa to Australia., 60: 1405-7 | |  |  |
| 236 | Chiou CS, Wei HL, Mu JJ et al, 2013, Journal of clinical microbiology, Salmonella enterica serovar Typhi variants in long-term carriers., 51: 669-72 | |  |  |
| 237 | Loharikar A, Newton A, Rowley P et al, 2012, Clinical infectious diseases : an official publication of the Infectious Diseases Society of America, Typhoid fever outbreak associated with frozen mamey pulp imported from Guatemala to the western United States, 2010., 55: 61-6 | |  |  |
| 238 | Judd MC, Grass JE, Mintz ED, Bicknese A, Mahon BE, 2015, Emerging infectious diseases, Salmonella enterica Paratyphi A Infections in Travelers Returning from Cambodia, United States., 21: 1089-91 | |  |  |
| 239 | Clarke K.R., Kanyanga M.K.. Musenga E et al., 2012, , Outbreak of multi-drug resistant salmonella Typhi, lusaka, zambia 2011-2012., 87: 318 | |  |  |
| 240 | ProMED-mail. Typhoid Fever in Mufulira, Zambia http://www.promedmail.org. [cited ProMED-mail archive:20120110.1005781] | |  |  |
| 241 | Tarupiwa A. Tapera S, Mtapuri-Zinyowera S et al, 2015, BMC research notes, Evaluation of TUBEX-TF and OnSite Typhoid IgG/IgM Combo rapid tests to detect Salmonella enterica serovar Typhi infection during a typhoid outbreak in Harare, Zimbabwe., 8: 50 | |  |  |
| 242 | ProMED-mail. Typhoid Fever in Ba, Fiji http://www.promedmail.org. [cited ProMED-mail archive:20120317.1073326] | |  |  |
| 243 | ProMED-mail. Typhoid Fever in Nanoko, Fiji http://www.promedmail.org. [cited ProMED-mail archive:20120110.1005781] | |  |  |
| 244 | ProMED-mail. Typhoid Fever in Viti Levu, Fiji http://www.promedmail.org. [cited ProMED-mail archive:20120317.1073326] | |  |  |
| 245 | ProMED-mail. Typhoid Fever in Himachal Pradesh, India http://www.promedmail.org. [cited ProMED-mail archive:20120212.1039842] | |  |  |
| 246 | ProMED-mail. Typhoid Fever in Karnataka, India http://www.promedmail.org. [cited ProMED-mail archive:20120317.1073326] | |  |  |
| 247 | Saitoh T, Morita M, Shimada T et al, 2016, Epidemiology and infection, Increase in paratyphoid fever cases in Japanese travellers returning from Cambodia in 2013., 144: 602-6 | |  |  |
| 248 | Hendriksen RS, Leekitcharoenphon P, Mikoleit M et al, 2015, Journal of clinical microbiology, Genomic dissection of travel-associated extended-spectrum-beta-lactamase-producing Salmonella enterica serovar typhi isolates originating from the Philippines: a one-off occurrence or a threat to effective treatment of typhoid fever?., 53: 677-80 | |  |  |
| 249 | ProMED-mail. Typhoid Fever in Alegria, Philippines http://www.promedmail.org. [cited ProMED-mail archive:20120310.1065939] | |  |  |
| 250 | ProMED-mail. Typhoid Fever in Leyte, Philippines http://www.promedmail.org. [cited ProMED-mail archive:20120110.1005781] | |  |  |
| 251 | ProMED-mail. Typhoid Fever in Tuburan, Philippines http://www.promedmail.org. [cited ProMED-mail archive:20120310.1065939] | |  |  |
| 252 | ProMED-mail. Typhoid Fever in Malie, Samoa http://www.promedmail.org. [cited ProMED-mail archive:20120321.1076101] | |  |  |
| 253 | ProMED-mail. Typhoid Fever in Upolo, Samoa http://www.promedmail.org. [cited ProMED-mail archive:20120321.1076101] | |  |  |
| 254 | Chiou CS, Wei HL, Mu JJ et al, 2013, Journal of clinical microbiology, Salmonella enterica serovar Typhi variants in long-term carriers., 51: 669-72 | |  |  |
| 255 | ProMED-mail. Typhoid Fever in Tongatapu, Tonga http://www.promedmail.org. [cited ProMED-mail archive:20120317.1073326] | |  |  |
| 256 | ProMED-mail. Typhoid Fever in Kabwe, Zambia http://www.promedmail.org. [cited ProMED-mail archive:20120420.1107274] | |  |  |
| 257 | ProMED-mail. Typhoid Fever in American Samoa http://www.promedmail.org. [cited ProMED-mail archive:20130618.1779531] | |  |  |
| 258 | ProMED-mail. Typhoid Fever in Phnom Penh, Cambodia http://www.promedmail.org. [cited ProMED-mail archive:20130928.1969953] | |  |  |
| 259 | ProMED-mail. Typhoid Fever in Kasai Occidental, Democratic Republic of Congo http://www.promedmail.org. [cited ProMED-mail archive:20130513.170163] | |  |  |
| 260 | ProMED-mail. Typhoid Fever in Taveuni, Fiji http://www.promedmail.org. [cited ProMED-mail archive:20130606.1756079] | |  |  |
| 261 | ProMED-mail. Typhoid Fever in Gujarat, India http://www.promedmail.org. [cited ProMED-mail archive:20130508.1697417] | |  |  |
| 262 | ProMED-mail. Typhoid Fever in Wagoora, India http://www.promedmail.org. [cited ProMED-mail archive:20130606.1756079] | |  |  |
| 263 | Feasey NA, Masesa C, Jassi C et al2015, Clinical infectious diseases : an official publication of the Infectious Diseases Society of America, Three Epidemics of Invasive Multidrug-Resistant Salmonella Bloodstream Infection in Blantyre, Malawi, 1998-2014., 61 Suppl 4: S363-71 | |  |  |
| 264 | ProMED-mail. Typhoid Fever in Malawi http://www.promedmail.org. [cited ProMED-mail archive:20130513.170163] | |  |  |
| 265 | Salwani H., Mohd Nuramin A., Fauziah M.N. et al 2014, , Genetic relationship and correspondence of Salmonella Typhi isolated from water samples in typhoid outbreak localities with food handlers and contact by using Pulsed Field Gel Electrophoresis (PFGE)., 4: 242 | |  |  |
| 266 | ProMED-mail. Typhoid Fever in Oas, Philippines http://www.promedmail.org. [cited ProMED-mail archive:20130618.1779531] | |  |  |
| 267 | Burki T, 2013, The Lancet. Infectious diseases, Infectious diseases in Malian and Syrian conflicts., 13: 296-7 | |  |  |
| 268 | ProMED-mail. Typhoid Fever in Central Province, Zambia http://www.promedmail.org. [cited ProMED-mail archive:20130508.1697417] | |  |  |
| 269 | Verma S, Sharma V, Mokta K et al, 2016, Indian journal of medical microbiology, Outbreak of enteric fever due to Salmonella Paratyphi A variety durazzo (2,12:a:-) in a hilly region of North India: A report of 43 cases., 34: 387-9 | |  |  |
| 270 | Roy JS, Saikia L, Medhi M, Tassa D, 2016, The Indian journal of medical research, Epidemiological investigation of an outbreak of typhoid fever in Jorhat town of Assam, India., 144: 592-596 | |  |  |
| 271 | Kobayashi T, Kutsuna S, Hayakawa K, Kato Y, Ohmagari N, Uryu H, Yamada R, Kashiwa N, 2016, The American journal of tropical medicine and hygiene, Case Report: An Outbreak of Food-Borne Typhoid Fever Due to Salmonella enterica Serotype Typhi in Japan Reported for the First Time in 16 Years., 94: 289-91 | |  |  |
| 272 | ProMED-mail. Typhoid Fever in Saptari, Nepal http://www.promedmail.org. [cited ProMED-mail archive:20140708.2595861] | |  |  |
| 273 | ProMED-mail. Typhoid Fever in Harare, Zimbabwe http://www.promedmail.org. [cited ProMED-mail archive:20140722.2626571] | |  |  |
| 274 | ProMED-mail. Typhoid Fever in Bua, Fiji http://www.promedmail.org. [cited ProMED-mail archive:20150122.3111081] | |  |  |
| 275 | ProMED-mail. Typhoid Fever in Sindhupalchok, Nepal http://www.promedmail.org. [cited ProMED-mail archive:20150726.3528925] | |  |  |
| 276 | ProMED-mail. Typhoid Fever in Damascus, Syria http://www.promedmail.org. [cited ProMED-mail archive:20150821.3591694] | |  |  |
| 277 | GIDEON online. Outbreak-> 2015->Kampala- Wakiso- Mukono districts, Uganda. 2015. | |  |  |
| 278 | Hancock-Allen J, Cronquist AB, Peden J, Adamson D, Corral N, Brown K, 2016, MMWR. Morbidity and mortality weekly report, Notes from the Field: Typhoid Fever Outbreak Associated with an Asymptomatic Carrier at a Restaurant - Weld County, Colorado, 2015., 65: 606-7 | |  |  |
| 279 | ProMED-mail. Typhoid Fever in Harare, Zimbabwe http://www.promedmail.org. [cited ProMED-mail archive:20160318.410372] | |  |  |
| 280 | | | Abade A, Eidex R, Maro A, Gratz J, Liu J, Kiwelu I et al. Use of TaqMan Array Cards to Screen Outbreak Specimens for Causes of Febrile Illness in Tanzania. The American Journal of Tropical Medicine and Hygiene. 2018;98(6):1640-1642. | |
| 281 | | | Nahimana M, Ngoc C, Olu O, Nyamusore J, Isiaka A, Ndahindwa V et al. Knowledge, attitude and practice of hygiene and sanitation in a Burundian refugee camp: implications for control of a Salmonella typhi outbreak in 2016. Pan African Medical Journal. 2017;28. | |
| 282 | | | Sambandamurthy V, Purighalla S, Esakimuthu S, Reddy M, Seth T, Patil S et al. Investigation into a community outbreak of Salmonella Typhi in Bengaluru, India. Indian Journal of Medical Research. 2017;146(7):15. | |
| 283 | | | Qamar F, Yousafzai M, Khalid M, Kazi A, Lohana H, Karim S et al. Outbreak investigation of ceftriaxone-resistant Salmonella enterica serotype Typhi and its risk factors among the general population in Hyderabad, Pakistan: a matched case-control study. The Lancet Infectious Diseases. 2018;18(12):1368-1376. | |
| 284 | | | ProMED-mail. Typhoid Fever in Auckland http://www.promedmail.org. [cited ProMED-mail archive:20170107.4749025] | |
| 285 | | | ProMED-mail. Typhoid Fever in Zambia http://www.promedmail.org. [cited ProMED-mail archive:20170524.5059531] | |
| 286 | | | ProMED-mail. Typhoid Fever in Syria http://www.promedmail.org. [cited ProMED-mail archive:20170523.5057372] | |
| 287 | | | ProMED-mail. Typhoid Fever in Tonga http://www.promedmail.org. [cited ProMED-mail archive:20170414.4971570] | |
| 288 | | | Bano-Zaidi M, Aguayo-Romero M, Campos F, Colome-Ruiz J, Gonzalez M, Piste I et al. Typhoid fever outbreak with severe complications in Yucatan, Mexico. 2019. | |
| 289 | | | ProMED-mail. Typhoid Fever in Guatemala http://www.promedmail.org. [cited ProMED-mail archive:20170809.5238178] | |
| 290 | | | ProMED-mail. Typhoid Fever in Europe http://www.promedmail.org. [cited ProMED-mail archive:20170927.5345228] | |
| 291 | | | ProMED-mail. Typhoid Fever in India http://www.promedmail.org. [cited ProMED-mail archive:20170808.5235812] | |
| 292 | | | ProMED-mail. Typhoid Fever in Korea http://www.promedmail.org. [cited ProMED-mail archive:20170823.5269596] | |
| 293 | | | ProMED-mail. Typhoid Fever in Guyana http://www.promedmail.org. [cited ProMED-mail archive:20170914.5316793] | |
| 294 | | | ProMED-mail. Typhoid Fever in Harare, Zimbabwe http://www.promedmail.org. [cited ProMED-mail archive:20170107.4749025] | |
| 295 | | | ProMED-mail. Typhoid Fever in Ohio http://www.promedmail.org. [cited ProMED-mail archive:20171006.5364847] | |
| 296 | | | ProMED-mail. Typhoid Fever in Fiji http://www.promedmail.org. [cited ProMED-mail archive:20171011.5374145] | |
| 297 | | | ProMED-mail. Typhoid Fever in South Africa http://www.promedmail.org. [cited ProMED-mail archive:20171203.5479456] | |
| 298 | | | ProMED-mail. Typhoid Fever in El Salvador http://www.promedmail.org. [cited ProMED-mail archive:20180618.5860604] | |
| 299 | | | ProMED-mail. Typhoid Fever in Syria http://www.promedmail.org. [cited ProMED-mail archive:20180323.5706324] | |
| 300 | | | ProMED-mail. Typhoid Fever in Massachusetts http://www.promedmail.org. [cited ProMED-mail archive:20180510.5792030] | |
| 301 | | | ProMED-mail. Typhoid Fever in Zimbabwe http://www.promedmail.org. [cited ProMED-mail archive:20180814.5964791] | |
| 302 | | | ProMED-mail. Typhoid Fever in India http://www.promedmail.org. [cited ProMED-mail archive:20181008.6077839] | |
| 303 | | | ProMED-mail. Typhoid Fever in Fiji http://www.promedmail.org. [cited ProMED-mail archive:20181207.6191941] | |
